# Biochemical Properties and Atomic Resolution Structure of a Proteolytically Processed β-Mannanase from Cellulolytic *Streptomyces* sp. SirexAA-E

**DOI:** 10.1371/journal.pone.0094166

**Published:** 2014-04-07

**Authors:** Taichi E. Takasuka, Justin F. Acheson, Christopher M. Bianchetti, Ben M. Prom, Lai F. Bergeman, Adam J. Book, Cameron R. Currie, Brian G. Fox

**Affiliations:** 1 Great Lakes Bioenergy Research Center, University of Wisconsin –Madison, Madison, Wisconsin, United States of America; 2 Department of Biochemistry, University of Wisconsin –Madison, Madison, Wisconsin, United States of America; 3 Department of Bacteriology, University of Wisconsin –Madison, Madison, Wisconsin, United States of America; Monash University, Australia

## Abstract

β-mannanase SACTE_2347 from cellulolytic *Streptomyces* sp. SirexAA-E is abundantly secreted into the culture medium during growth on cellulosic materials. The enzyme is composed of domains from the glycoside hydrolase family 5 (GH5), fibronectin type-III (Fn3), and carbohydrate binding module family 2 (CBM2). After secretion, the enzyme is proteolyzed into three different, catalytically active variants with masses of 53, 42 and 34 kDa corresponding to the intact protein, loss of the CBM2 domain, or loss of both the Fn3 and CBM2 domains. The three variants had identical N-termini starting with Ala51, and the positions of specific proteolytic reactions in the linker sequences separating the three domains were identified. To conduct biochemical and structural characterizations, the natural proteolytic variants were reproduced by cloning and heterologously expressed in *Escherichia coli*. Each SACTE_2347 variant hydrolyzed only β-1,4 mannosidic linkages, and also reacted with pure mannans containing partial galactosyl- and/or glucosyl substitutions. Examination of the X-ray crystal structure of the GH5 domain of SACTE_2347 suggests that two loops adjacent to the active site channel, which have differences in position and length relative to other closely related mannanases, play a role in producing the observed substrate selectivity.

## Introduction

Mannan, one of the major hemicelluloses in higher plants, is primarily composed of β-1,4 linked D-mannose units. Structural variations of mannan are classified according to the types of sugars that are incorporated into the mannan chain. For example, galactomannan contains D-galactose attached to the mannan chain *via* α-1, 6 linkages [1], [2], while glucomannan has the chain substituted with D-glucose. Furthermore, galactoglucomannan, which is prevalent in pine wood, has glucose incorporated into the mannan chain and galactosyl branching. In addition to the incorporation of sugar, the C2 and C3 hydroxyl groups of both mannosyl and glucosyl unit of mannan are frequently acetylated [2]. Hydrogen bonding interactions between the galactosyl branches and the mannan chain along with the physical association of hemicellulose with cellulose makes deconstruction of hemicellulose and other plant cell wall polysaccharides a formidable task [3]. Thus, enzymatic hydrolysis of mannan-containing polymers is essential for deconstruction of plant cell wall, particularly softwoods such as pine.

Mannan hydrolysis is carried out by free-living soil microorganisms [4]–[6], including numerous species in the *Streptomyces* genus. In order to utilize mannan-containing polysaccharides, these organisms secrete β-mannanases, β-mannosidases, α-1,6 galactosidases, and acetylmannan esterases in addition to other polysaccharide-degrading enzymes [7]–[9]. Synergy between enzymes of different functions enables efficient deconstruction of biomass.

Recently, we described the cellulolytic and hemicellulolytic capability of *Streptomyces* sp. SirexAA-E, an aerobic microbe that is a prominent member of a bacterial/fungal symbiotic community associated with the invasive pinewood-boring wasp *Sirex noctilio* [10], [11]. Proteomic analysis showed that when SirexAA-E is grown on biomass substrates, it secretes numerous endo- and exocellulases, xylanases, polysaccharide monooxygenases, a caffeoyl-CoA dioxygenase [12], and a single β-mannanase, SACTE_2347 [11]. The specific activity of the SirexAA-E secretome was comparable to that of Spezyme CP (Genencor, International Inc, NY, USA), an early generation commercial cellulase cocktail prepared from *Trichoderma reesei* Rut-C30. Moreover, the xylan- and mannan-hydrolytic activities of the SirexAA-E secretome were higher than those detected in Spezyme CP. SACTE_2347 was identified in secretomes produced when SirexAA-E was grown on cellobiose, cellulose, or various pretreated biomass samples. Interestingly, polypeptides with masses of 53, 42 and 34 kDa were identified by mass spectrometry fractions of the SirexAA-E secretome containing the highest mannanase activity, presumably generated by extracellular proteolytic processing. Since the hemicellulosic fraction of pine wood is greatly enriched in mannan-containing polysaccharides, the properties of enzymes participating in hemicellulosic deconstruction was of interest.

Here we report that the three different variants of β-mannanase from SirexAA-E are derived from SACTE_2347. As all three are abundant in the secreted proteome, we determined the catalytic properties of each. All three variants were capable of hydrolyzing mannan, glucomannan, and galactomannan, which are the three different forms of mannan predominant in pine wood. The full-length enzyme, which contains the CBM2 domain, has modestly improved catalytic efficiency for reaction with pure β-D-mannan and ionic liquid-pretreated pine wood. Additionally, we report the atomic resolution (1.06 Å) crystal structure of the GH5 domain of SACTE_2347, which revealed a unique arrangement of two loops adjacent to the active site channel. The product distributions obtained from exhaustive hydrolysis of galactomannan are interpreted in light of the structural constraints presented by this newly observed arrangement of loops. A potential role of this enzyme in the *Sirex*-microbe symbiotic community during the invasive attack on pine wood is also considered.

## Results

### Bioinformatics

**Figure 1:**
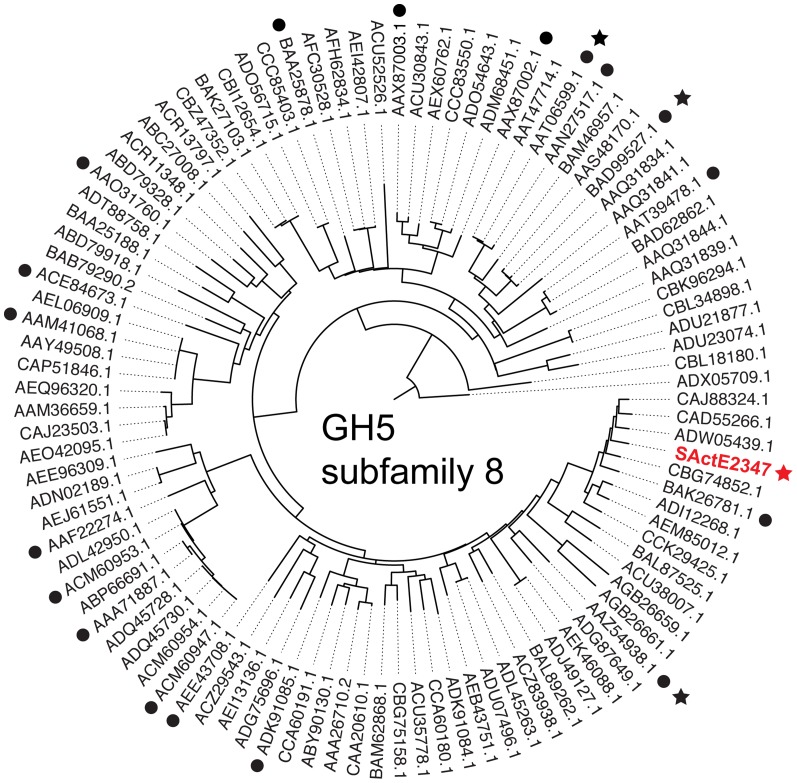
Phylogeny of GH5 subfamily 8 including SACTE_2347. The phylogenic tree was constructed from all sequences assigned to the GH5 subfamily 8. Names shown are GenBank accession codes. Black circles indicate enzymes that have been experimentally verified to exhibit β-mannanase activity; black stars indicate three enzymes whose structures have been determined besides SACTE_2347. These are: *Thermomonospora fusca*, AAZ54938.1, PDB 1BQC, 2MAN, 3MAN; *Bacillus* sp. N16-5, AAT06599.1, PDB 2WHJ, 2WJL, 3JUG; *Bacillus* sp. JAMB-602, BAD99527.1, PDB 1WKY.

**Figure 2:**
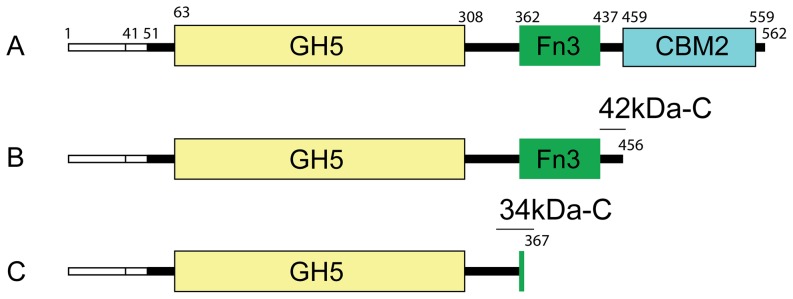
Schematics of the domain structure in three variants of SACTE_2347. A, SACTE_2347_FL; B, SACTE_2347_42kDa; C, SACTE_2347_34kDa. Residues 1–41 correspond to the annotated twin arginine translocation peptide. Residue 51 corresponds to the experimentally determined N-terminus in the three variants. The GH5 domain spans residues 63–308, and boundaries of the Fn3 and CBM2 domains are as indicated. The location of C-terminal tryptic-digest polypeptides for each variant is shown (GH5, 34kDa-C, Fn3, 42kDa-C, CBM2; also shown in Table 2 and Figure S1).

The SirexAA-E genome encodes three GH5 family enzymes: SACTE _0482 (subfamily 2, with cellulase activity detected); SACTE _5461 (subfamily 19, no functional assignment); and SACTE_2347, which is a member of subfamily 8. Of the 101 genes currently assigned to GH5 subfamily 8, only 18 have been experimentally shown to possess β-1,4-mannanase activity (black dots in Figure 1). SACTE_2347 is a three-domain protein (Figure 2) that consists of a GH5 catalytic domain (residues 63–308), a fibronectin type-III domain (Fn3, residues 362–437), and a CBM2 (residues 459–559) that are connected by two short linkers (residues 309–361 and residues 438–458). The gene also encodes a twin-arginine translocation signal peptide (residues 1–41), which targets fully folded proteins for secretion [13], and is frequently associated with cellulolytic proteins secreted by SirexAA-E [11].

### Forms of SACTE_2347 in cellulolytic secretomes

**Figure 3:**
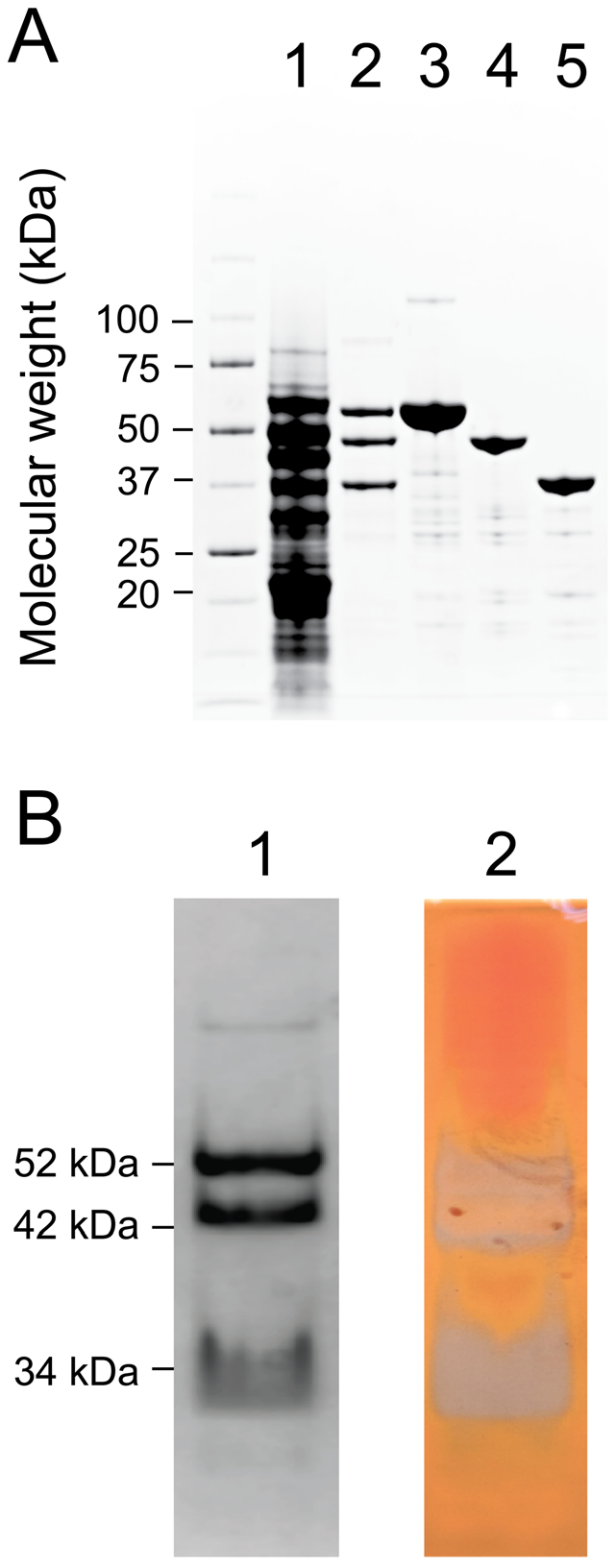
PAGE analysis of SACTE_2347 preparations. A, Lane 1, *Streptomyces* sp. SirexAA-E secretome; lane 2, fraction from the ion exchange separation that showed the maximal mannanase activity; lane 3, recombinant SACTE_2347_FL purified from *E. coli* BL21(DE3); lane 4, recombinant SACTE_2347_42kDa; lane 4 recombinant SACTE_2347_34kDa. B, in-gel determination of mannan-degrading activity. Lane 1, native PAGE with Coomassie Blue staining of the fraction from A, lane 2 performed with mannan included in the gel; lane 2, Congo Red staining of the gel. Regions of the gel that contain polysaccharide bind the dye and appear orange; regions of the gel containing enzyme activity no longer contain polysaccharide and so have a grey-colored appearance.

The preparation of cellulolytic secretomes from SirexAA-E, ion exchange separation, and preliminary determination of various enzymatic activities has been reported [11]. Here we provide an in-depth biochemical and structural characterization of SACTE_2347, the only secreted mannanase from this highly cellulolytic organism. All fractions from ion exchange that exhibited mannanase activity contained three dominant polypeptides with masses of ∼34 kDa, ∼42 kDa and ∼52 kDa (Figure 3A). To qualitatively screen the mannan-degrading activities of these polypeptides, an in-gel assay was carried out with β-1,4 D-mannan. After electrophoretic separation and time for the enzymatic reaction to proceed, Congo Red staining showed that all three polypeptides had β-mannanase activity (Figure 3B). This suggested that the three polypeptides were derived from full-length SACTE_2347. Since prolonged incubation of the secretome increased the proportion of the two smaller polypeptides at the expense of the larger, this proteolysis was a property of the secretome, which is known to contain several proteases [11].

In order to identify the N- and C-terminal amino acid sequences of the three SACTE_2347 polypeptides, amino terminal sequencing and mass spectrometry were performed (Figure 2, Table 1 and Figure S1). All three SACTE_2347 variants had the same N-terminal sequence of Ala51-Ala52-Gly53-Leu54. Interestingly, this sequence does not correspond to that immediately following the predicted twin arginine translocation signal in SACTE_2347, which is Asp42-Leu43-Pro44-Gln45. Whether this difference is due to reaction of the transport peptidase at a position different than that predicted by bioinformatics or is due to the presence of another protease in the secretome cannot be determined at this time.

**Table 1:** Peptide sequences identified by mass spectrometry.

Peptide^a^	Mass (Da)	*m/z* [M+H]^2+^,^b^	Peptide sequence
42kDa-C	1448.7	725.34	Ser441-GTVSVTTDEGGSVP-Gly456
34kDa-C	2174	1088	Glu344-ATVFGGGQGGGDTEAPTAPGTP-Thr367
GH5	2658.2	1330.12	Ala201-AGFAHTIMVDAPNWGQDWEGVM-Arg224
Fn3	2608.4	1305.2	Val405-ASSAATSVTVTGLSAGTAYSFAVYA-Arg431
CBM2	1757.9	879.96	Val465-IGEWPGGFQGEITL-Arg480

a The full protein sequence of SACTE_2347, annotated with the positions of these peptides is found in Figure S2. The names of peptides are also used in Figure 2.

b Observed *m/z*.

Analysis of the mass data revealed that the largest protein corresponded to the sequence from Ala51 to Ala562, where the intact *m/z* = 52581 Da [M+H]^+^ is in agreement with the theoretical molecular weight of 52564 Da. This protein also yielded diagnostic tryptic peptides from the GH5, Fn3 and CBM2 domains (*m/z* = 1330.12, 1305.2 and 879.96, respectively). The intermediate sized protein (intact *m/z* = 42373 Da [M+H]^+^ and theoretical molecular weight of 42355 Da) yielded diagnostic tryptic peptides from both GH5 and Fn3 domains (*m/z* = 1330.12 and 1305.2, respectively), while the smallest protein (intact *m/z* = 33,875 Da [M+H]^+^, and theoretical molecular weight of 33822 Da) yielded a diagnostic tryptic peptide from only the GH5 domain (*m/z* = 1330.12). Furthermore, C-terminal peptides for the intermediate and smallest sized protein were also detected (Table 1 and Figure S1). A peptide with *m/z* = 725.34 [M+H]^2+^ was identified corresponding to the sequence from Ser441 to Gly456 at the C-terminus of the intermediate-sized protein. This peptide lies in the linker region between the Fn3 and CBM2 domains. Furthermore, a peptide with *m/z* = 1088 [M+H]^2+^ was identified from the C-terminus of the smallest protein, corresponding to the sequence from Glu344 to Thr367. This peptide includes the linker region between the GH5 and Fn3 domains, and also the first six residues from the Fn3 domain. No other peptides with masses consistent with assignment to the C-terminus were detected. The combination of masses of the different polypeptides and sequence assignments for the C-terminal peptides confirm that the proteolytic processing of SACTE_2347 occurs at specific positions in the linker regions between the domains.

In order to better understand the function of SACTE_2347 and its proteolytically processed forms, plasmids encoding the protein sequences identified by N-terminal sequencing and mass spectrometry were produced, and the corresponding proteins were expressed in *Escherichia coli*, purified, and assayed with a panel of different substrates. These three recombinant proteins are designated SACTE_2347_FL (residues 51–562), SACTE_2347_42kDa (residues 51–456), and SACTE_2347_34kDa (residues 51–367).

### Crystal Structure

Data collection, refinement, and model statistics are summarized in Table 2. Crystal of His-tagged SACTE_2347_34kDa belonged to the P2_1_2_1_2 space group and contained one monomer per asymmetric unit. The 1.06 Å structure of SACTE_2347_32kDa has a His tag bound in the active site. This interaction is propagated throughout the crystal lattice, and likely contributes to the high degree of order observed.

**Table 2:** Summary of crystal parameters, data collection, and refinement statistics.

	SACTE_2347_34KDa
**Crystal parameters**	
Space group	P2_1_2_1_2
Unit-cell parameters (Å)	62.81, 102.36, 45.35
**Data collection statistics**	
Resolution range (Å)	36.77–1.06 (1.09–1.06)
Completeness (%)	97.7 (95.2)
R_merge_ *	0.063 (0.31)
Redundancy	3.5 (2.8)
Mean I/sigma (I)	12.8 (3.17)
Wilson B factor (Å^2^)	8.4
**Refinement and model statistics**	
Resolution range (Å)	36.77–1.06 (1.09–1.06)
No. of reflections (work/test)	128004/2000
R_cryst_ §	0.118 (0.158)
R_free_	0.132 (0.175)
RMSD bonds (Å)	0.007
RMSD angles (°)	1.232
B factor (Å^2^)	12.65
No. of protein atoms	2971
No. of waters	507
**Ramachandran plot (%)**	
Favorable region	97.76
Additional allowed region	1.93
Disallowed region	0.32
**PDB ID**	4FK9

*R_merge_ = ∑_h_ ∑_i_ | I_i_ (h)−<I(h)>|/∑_h_∑_i_ I_i_(h), where I_i_(h) is the intensity of an individual measurement of the reflection and <I(h)> is the mean intensity of the reflection.

§ R_cryst_ = ∑_h_ ||F_obs_|−|F_calc_||/∑_h_ |F_obs_|, where F_obs_ and F_calc_ are the observed and calculated structure-factor amplitudes, respectively.

¶ R_free_ was calculated as R_cryst_ using 1.5% of randomly selected unique reflections that were omitted from the structure refinement.

Values in parentheses are for the highest resolution shell.

### Active Site Channel

**Figure 4:**
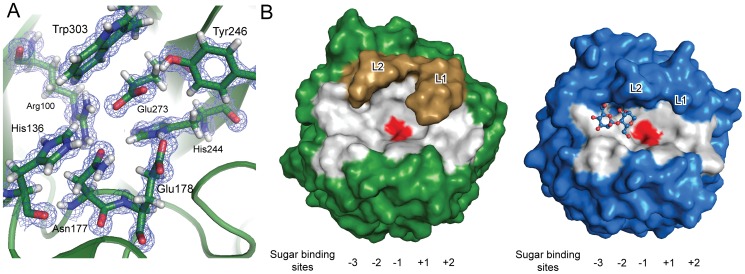
Atomic resolution structure of SACTE_2347. A, 2Fo-Fc electron density map of eight conserved residues in the GH5 family, contoured at 1.3 σ. Hydrogen atoms were included in the refinement of the high-resolution data. B, Comparison of the active site channels of SACTE_2347 (green) and TfManA (blue). Residues that form the surface of the channel are highlighted in gray, and the positions of loops L1 and L2 are indicated. Positions of the catalytic residues (Glu178 and Glu273 in SACTE_2347_34kDa) are shown in red. Mannobiose observed in the −3 and −2 subsites of the TfManA structure is shown as ball and sticks.

Despite the high degree of amino acid sequence diversity, the GH5 family is defined by eight strictly conserved residues [14]. In SACTE_2347, these eight residues correspond to Arg100, His136, Asn177, Glu178, His244, Tyr246, Glu273, and Trp303 (Figure 4), with Glu178 and Glu273 serving as the catalytic acid/base and nucleophile, respectively. The conservation of active site residues and the distances between the catalytic acid and base suggests that SACTE_2347 hydrolyzes glycosidic bonds with retention of the anomeric configuration through a double displacement mechanism [15], [16].

An extended solvent-exposed oligosaccharide binding channel that spans the face of the (β/α)_8_-barrel is a prominent features of GH5 enzyme architecture (Figure 4B). Structural differences along this channel provide the molecular basis for the remarkable diversity of activities that are observed among GH5 members. While six subsites (−4, −3, −2, −1, +1, and +2) were identified in the mannotriose-bound TfManA structure (PDB ID 3MAN, 0.35 Å rmsd [17]), conserved structural features between the two enzymes and the shorter length of the SACTE_2347_34kDa oligosaccharide binding channel (Figure 4B) allowed us to positively identify five potential mannose-binding subsites (−3, −2, −1, +1 and +2) [18]. The glycosidic bond placed between the −1 and +1 sites is at the position of hydrolysis. Several aromatic and polar residues positioned along this channel provide a platform for sugar binding. For example, Trp30 of TfManA forms a stacking interaction with a mannose at the −3 subsite, and Trp80 from SACTE_2347_34kDa aligns with this residue. Residues that form the −3 and −2 subsites in the TfManA mannotriose-bound structure (e.g., Tyr81, Trp109, His136, Trp303, and Asn308) are also identical or highly conserved in SACTE_2347_34kDa and all other GH5 subclade 8 enzymes, suggesting that binding at the −3 and −2 subsites is conserved within this family.

The SACTE_2347_34kDa and TfManA structures differ substantially in lengths and positions of two loops, L1 and L2, that form part of the active site channel (Figure 4B). Additional analysis on the positioning of comparable loops in other GH5 enzymes is provided in Table S1. In summary, among the 19 structures of GH5 enzymes that were solved with and without bound ligands including His tags, the position of the loops that compose the substrate bind channel do not change regardless of whether oligosaccharides or His tags are present or not. Thus, the presence of the bound His tag is not likely to have a significant impact on the position of L1 and L2 in the SACTE_2347_34KDa structure. In the following, we present an analysis of the positioning of loops L1 and L2 in SACTE_2347 relative to the catalytic residues (Glu178 and Glu273, red surface in Figure 4B), and then consider how this arrangement permits reaction with galactomannan typically found in pine wood.

Figure 4B shows that L1 loop residues (275–284) in the SACTE_2347_34kDa adopts an extended conformation that might alter substrate accessibility at the +1 and +2 subsites relative to TfManA, which lacks a comparable loop (Figure S2). Interestingly, Tyr281 from SACTE_2347_34kDa is positioned at the tip of L1, where it could interact with a bound mannan chain sugar, but also provide steric exclusion to the placement of an α-1, 6 linkaged glucomannan unit into the +1 and perhaps the +2 subsites. Furthermore, L2 loop (residues 302–315) in the SACTE_2347_34kDa is two residues shorter and shifted toward the −3 site as compared to L2 loop of TfManA (Figure S2). In the structures of TfManA and other mannanases, the additional two residues in the L2 loop are part of a two-turn helix that protrudes into the active site channel. This feature might provide closer steric interaction at the −2 and −1 subsites, than those possible in SACTE_2347, which lacks this loop. Furthermore, L1 and L2 do not make contacts with each other in SACTE_2347_34kDa, but instead provide an opening between the two loops that is perpendicular to the main path of the channel (Figure 4B) and above the catalytic residues. The space between L1 and L2 is large enough to allow a branching sugar to bind in the −1 position. Moreover, the shorter and displaced position of L2 offers the possibility to accommodate a branching sugar at the −2 position of SACTE_2347_34kDa.

### Function of SACTE_2347

To test our hypothesis for substrate selectivity arising from the unique features in the SACTE_2347 structure, SACTE_2347 and its proteolytically processed forms were tested using a panel of different substrates. The SACTE_2347 variants did not react with any of seven fluorescent substrates tested, including MUM, a diagnostic analog for mannobiosidase activity. This result indicates that mannosyl groups must occupy at least three sugar binding subsites along the active site channel in order to achieve catalysis.

Reactions with insoluble polysaccharides were tested using the DNS assay to detect release of reducing sugars. The SACTE_2347 variants were able to hydrolyze β-1,4 D-mannan, acetylated glucomannan and locust bean gum, the latter of which contains on average one galactose branching unit for every 3–4 mannose units. No variant was active on cellulose, galactan or xylan. Table 3 lists the steady-state kinetic parameters for hydrolysis obtained with the different SACTE_2347 variants. These studies were conducted at pH 6.0 and 40°C, which was an optimum condition for the SACTE_2347 (Figure 3S), and the kinetic parameters were from short time assays (15 min) where only ∼1–2% of the total substrate present was hydrolyzed. Among the three variants, SACTE_2347_FL containing the CBM2 domain showed a modest increase in apparent *k*_cat_/*K*_M_ for mannan, acetylated glucomannan, and IL-pine relative to SACTE_2347_34kDa lacking the CBM2 domain. This relationship did not hold with locus bean gum, a structurally more complex substrate, where equivalent *k*_cat_/*K*_M_ values were observed with all three variants.

**Table 3:** Kinetic constants determined for SACTE_2347 variants.

	*k*_cat_	*k*_cat_	*K*_M,app_	*K*_M_	*k*_cat_/*K*_M_	*K*_cat_/*K*_M_	*V*_max_	*V*_max_
	(s^−1^)	error	(mg/mL)	error	s^−1^ (mL mg^−1^)	Error	(U/mg)	error
Mannan^a^								
SACTE_2347_34kDa	32	2	3	0.4	11	5	57	3
SACTE_2347_42kDa	33	1	3	0.2	11	5	47	2
SACTE_2347_FL	33	1	2	0.3	17	3	38	2
Acetylated glucomannan^b^								
SACTE_2347_34kDa	3	0.2	0.4	0.1	8	2	6	0.4
SACTE_2347_42kDa	4	0.3	0.3	0.1	13	3	5	0.4
SACTE_2347_FL	4	0.2	0.3	0.1	13	2	5	0.2
Locust bean gum^c^								
SACTE_2347_34kDa	41	3	2	0.4	21	8	73	3
SACTE_2347_42kDa	37	2	2	0.3	19	7	52	3
SACTE_2347_FL	41	2	2	0.2	21	10	47	2
IL-pine wood^d^								
SACTE_2347_34kDa	4	0.8	16	6	0.2	0.1	6	1
SACTE_2347_42kDa	5	1	21	6	0.2	0.2	6	1
SACTE_2347_FL	6	1	19	7	0.3	0.1	7	2

a Pure β-1,4 d-mannan.

b Acetylated glucomannan contain mannan (60%) and glucose (40%).

c Locust bean gum is a natural galactomannan with composition of ∼3.5 mannose per galactose.

d IL-pine has the following composition: 34% glucose; 9% xylose; 8% mannose; 4% arabinose, and 8% galactose.

SACTE_2347 did not hydrolyze cellulose, xylan and other polysaccharides described in the Materials and Methods, and likewise did not react with fluorogenic small molecule analogs.

**Figure 5:**
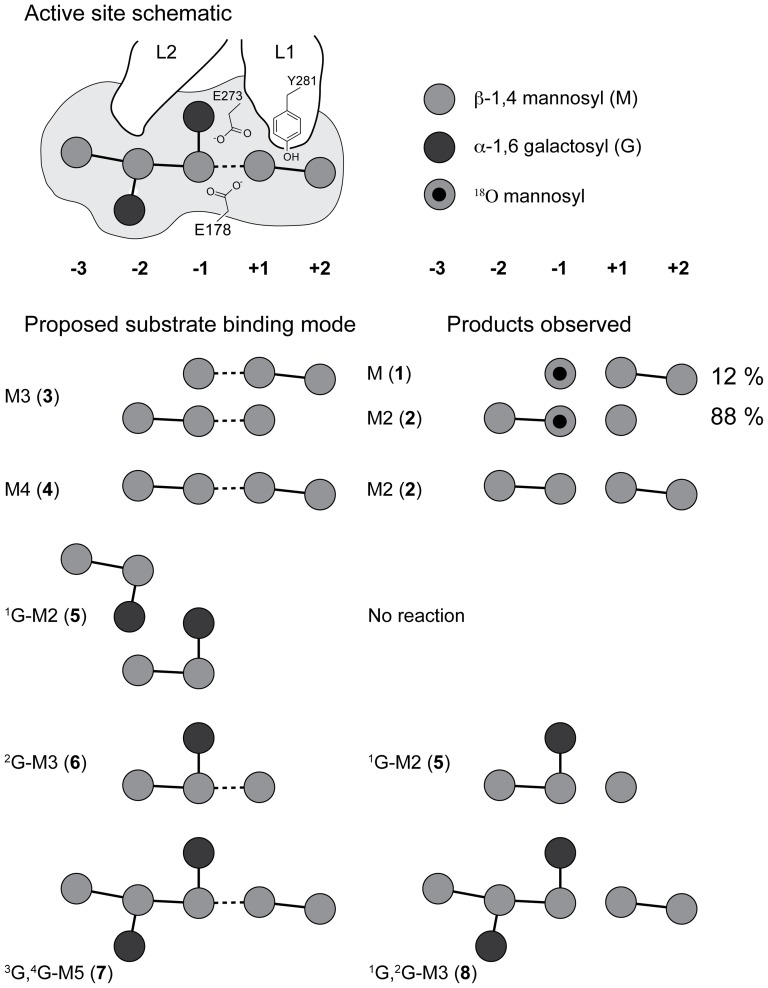
Schematic diagram of the binding subsites of SACTE_2347 correlated with reaction of purified oligomannosides and galactosyl-substituted oligomannosides. The active site schematic shows the positions of sugar binding subsites, the catalytic residues Glu178 and Glu272, and the position of loops L1 and L2. Mannosyl groups (grey circles) and galactosyl groups (black circles) of purified substrates studies are aligned in the −3 to +2 subsites under the schematic of the active site channel. Loop L1 blocks binding of a substituted mannosyl group in either the +1 of +2 subsites. The space between L1 and L2 allows placement of a substituted mannosyl group in the −1 subsite, while shortened L2 allows placement of a substituted mannosyl group into the −2 subsite. All reaction products can be rationalized to arise from hydrolysis of the glycosidic bond between the −1 and +1 subsites after accounting for steric interactions with L1 and L2.

In the following, the bold numbers shown below the structures of the substrates and products shown in Figure 5 will be used as identifiers. An HPLC analysis was performed to determine the end products from SACTE_2347-catalyzed reactions. SACTE_2347 did not hydrolyze mannobiose (**2**), but produced mannose (**1**) and **2** from mannotriose (**3**). These products may arise from binding of **3** across either the −2 to +1 or the −1 to +2 subsites. To better understand the binding mode of **3**, we performed reactions in buffer enriched with ^18^OH_2_ to trace the positioning of the substrate in the sugar binding subsites at the time of hydrolysis [19]. The relative ^18^O incorporation in **1** versus **2** was determined by ESI-TOF mass spectrometry, and ∼7.5-fold excess of ^18^O-mannobiose over ^18^O-mannose was observed, indicating a preference for occupation of the −1 to +2 subsites during catalysis (Figure 5).

In other reactions, **2** was the only product observed from mannotetraose (**4**). Thus, SACTE_2347 lacks β-1,4 mannosidase activity. This catalytic selectivity is also consistent with the lack of activity with the diagnostic fluorescent substrate MUM.

Several purified α1,6-linked galactosyl mannooligosaccharides were used to further correlate the reactivity with the positions of the L1 and L2 selectivity loops relative to the assigned sugar binding subsites (Figure 5). SACTE_2347 hydrolyzed 6^2^-α-D-galactosyl-mannotriose (**6**) into 6^1^-α-D-galactosyl-mannobiose (**5**) and **1**, and 6^3^,6^4^-α-D-galactosyl-mannopentaose (**7**) into 6^1^,6^2^-α-D-galactosyl-mannotriose (**8**) and **2**. SACTE_2347 did not further react with products **5** or **8**. In summary, SACTE_2347 reacted with purified mannan and galactosyl branched mannans to give **2**, and two branched oligosaccharides, **5** and **8**.

**Figure 6:**
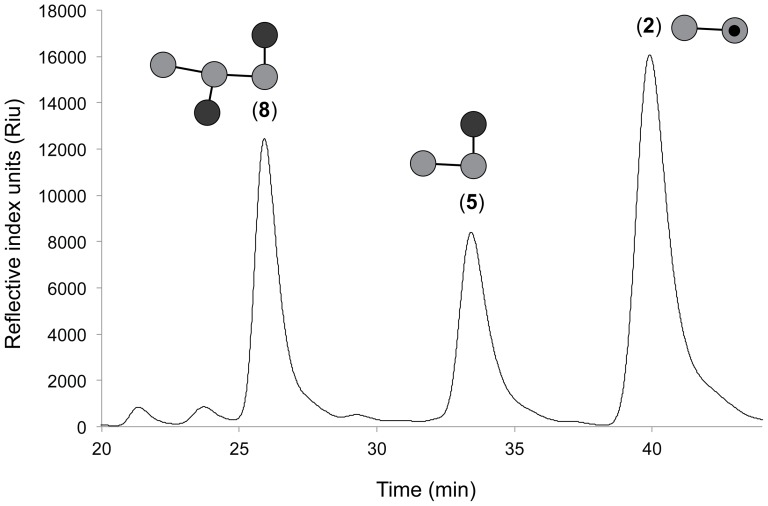
End products from exhaustive hydrolysis of locust bean gum by SACTE_2347 determined by HPLC. The three major products identified by comparison of elution times with purified commercial standards were ^1^G,^2^G-M3 (**8**), ^1^G-M2 (**5**), and M2 (**2**).

Locust bean gum is a natural product mannan containing α-1,6-linked galactosyl groups. According to the manufacturer, this material contains, on average, 1 galactosyl modification per 3.5 mannose units. Figure 6 show that the products obtained from exhaustive hydrolysis of locust bean gum by SACTE_2347 were **2**, **5**, and **8**. Among these three compounds, there are a total of 7 mannose units and 3 galactosyl units. After normalization using the integrated areas of the chromatograph peaks, the calculated relative proportion of mannose to galactose in the total final products was 3.7, which matches the value obtained by chemical hydrolysis reported by the manufacturer. According to the structural constraints established by L1 and L2, **8** accumulates as a non-reactive product, while **2** and **5** are too short to be further hydrolyzed.

Since pine wood is the preferred natural substrate for the *Sirex* symbiotic community, IL-pine and other biomass substrates were tested for reaction. The monocot substrates (corn stover and switchgrass) contain only a minor fraction of mannan, and so no significant reactivity was observed. All SACTE_2347 variants reacted with IL-pine, which contains ∼8% (w/w) of mannan polysaccharides, although the *k*_cat_ and *k*_cat_/*K*_M_ values were lower than for the purified polysaccharides.

**Figure 7:**
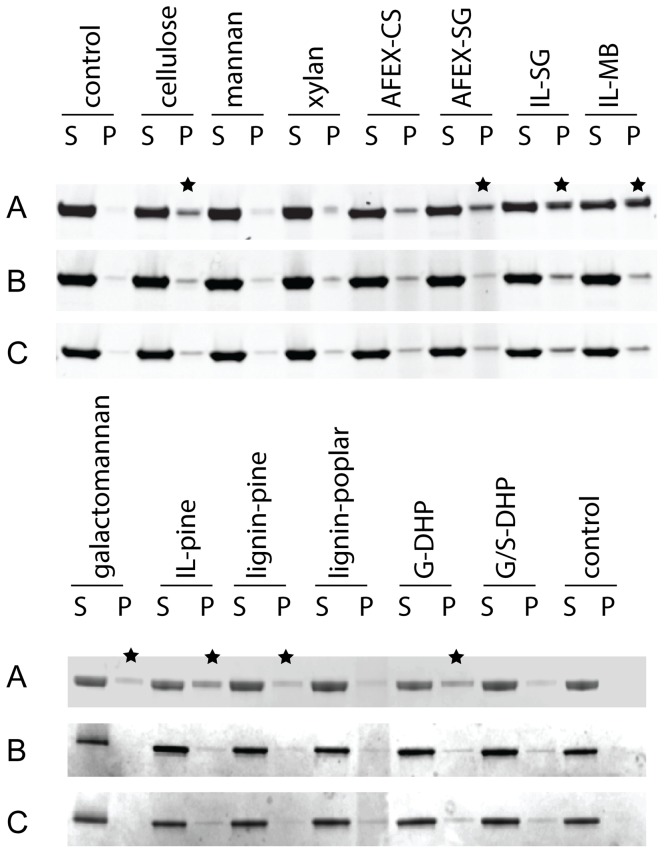
Insoluble substrate binding assays. SACTE_2347 variants were tested for binding to the insoluble substrates indicated using a pull-down format. A, SACTE_2347_FL. B, SACTE_2347_42kDa. C, SACTE_2347_34kDa. The presence of protein in the pellet fraction (P) indicates binding, as the control lane showed each variant was fully soluble in the conditions tested in the absence of an insoluble substrate. Black stars indicate the substrates for which binding was observed.

Given this result, it was of interest to determine the binding selectivity of the CBM2 domain in SACTE_2347. CBM2 domains have been experimentally demonstrated to bind to cellulose, xylan and chitin [20]–[22]. The equilibrium binding of each isoform was tested by using insoluble polysaccharide pull-down assays (Figure 7). The presence of the CBM2 domain in SACTE_2347_FL promoted binding to pure cellulose and galactomannan, whereas the two smaller isoforms lacking the CBM2 domain did not bind. Interestingly, none of the SACTE_2347 isoforms bound tightly enough to insoluble mannan to be detected by the pull-down assay. Binding was observed with AFEX-treated corn stover and switchgrass, which is consistent with the exposure to cellulose provided by this pretreatment [23]. The binding capacity with the IL-treated biomass and IL-pinewood increased as the biomass was changed from a grass to mixed biomass and then pine, possibly corresponding to a progressive increase in the galactomannan content. Furthermore, SACTE_2347_FL bound to the lignin from the pine and synthetic G-DHP lignin [24].

## Discussion

### GH5 Subfamily 8

The GH5 family is separated into 53 subfamilies, with each subfamily defined by sequence similarity, amino acid conservation, and experimentally determined biochemical properties [25]. In this study, we report biochemical and structural properties of SACTE_2347, a GH5 mannanase from the cellulolytic SirexAA-E. SACTE_2347 is a member of the GH5 subfamily 8 (Figure 1).

The majority of enzymes with β-1,4 mannanase activity have been assigned to either the GH5 or GH26 [17], [26]–[29], with one example from GH113 reported [30]. Although enzymes from these families hydrolyze mannan with retention of stereochemistry at the anomeric carbon, the families differ in their domain structures. Whereas GH5 mannanases are most often a combination of catalytic domains with a CBM and/or additional GH domains, GH26 mannanases are primarily single domain enzymes. The SirexAA-E genome does not encode a member of the GH26 or GH113 family. Consequently, the SACTE_2347 gene is the only annotated mannanase present in the SirexAA-E genome. This gene encodes a polypeptide consisting of GH5, Fn3, and CBM2 domains.

Differences in domain structures of cellulolytic enzymes provide the variation needed to deconstruct the diversity of polysaccharide structures present in plant cell walls [31]. For example, single domain mannanases have been proposed to more readily hydrolyze soluble, easily accessible to oligomannosaccharides without the potential complication of being adsorbed to insoluble polysaccharides (or other biomass constituents) through the presence of a CBM domain [27]. In contrast, multi-domain enzymes that contain a CBM might have increased activity on insoluble mannan by virtue of the ability of the CBM to more efficiently associate the catalytic domain to the substrate surface. Our results indicate that SACTE_2347 is proteolytically processed to yield three well-defined variants in the SirexAA-E secretome obtained from growth on biomass. All variants of SACTE_2347 retained the ability to hydrolyze insoluble mannan substrates, but did not react with galactan, xylan, or cellulose. Overall, there was little difference in the *k*_cat_/*K*_M_ observed for the three variants with the purified mannans, but SACTE_2347_FL, containing the CBM2 domain, had a 40–60% improvement in *k*_cat_/*K*_M_ for reaction with pure mannan and IL-pine, a galactomannan-enriched substrate (Table 3).

The SACTE_2347 CBM2 domain was bound to cellulose, galactomannan and various lignins analogs in pull-down assays. Thus, tight-binding interaction of SACTE_2347_FL with biomass likely occur *via* exposed regions of any of these polymers. It is also interesting that SACTE_2347_FL was bound to galactomannan, but not to pure D-mannan (composition of 97% mannose and 3% galactose). This result implies that galactosyl-branches on the mannan chain may have an important contribution to binding interactions with this CBM2.

### Structural basis for subsite selectivity

The SACTE_2347_32kDa structure has a His tag from a symmetry related monomer bound in the active site channel. Comparison with other structures of GH5 enzymes surprisingly revealed no significant differences in the positions of active site residues and adjacent loops regardless of whether ligands or His tags were bound in the active site (Table S1 and associated analysis). Although attempts to obtain structures from SACTE_2347 lacking the His tag or by soaking the tagged protein with various substrates and products were not successful, we used catalytic studies with purified of β-1,4 D-mannan, α-1,6 substituted mannans, and locust bean gum to provide further insight into the structural basis for selectivity of SACTE_2347 hydrolysis. For the catalytic studies, the His tag was removed.

Figure 5 provides a schematic of the SACTE_2347 active site channel showing the positions of the catalytic residues (Glu178 and Glu272), and correlates the proposed subsites used for substrate binding with the positions of selectivity-controlling loops L1 (including Y281) and L2, and the observed products. All SACTE_2347 variants were unable to hydrolyze **2** but did hydrolyze **3** and **4**. The preferential formation of ^18^O-mannobiose suggests that **3** must bind across the −2 to +1 subsites. This result is also consistent with the end product formation pattern determined in 6^1^-α-D-galactosyl-mannotriose. The exclusive hydrolysis of **4** to **2** indicates that **4** must bind across the −2 to +2 subsites, and again emphasizes the importance of occupying the −2 to +1 subsites. The extended configuration of L1, which includes Tyr281 at the tip of the loop, provides a potential steric mechanism for refining interactions with substrates. For example, a favorable interaction of Tyr281 with a mannosyl group bound in the active site channel would also disfavor placement of mannosyl groups with a branching sugar substitution into the +1 and +2 subsites.

Galactosyl mannooligosaccharides were used to further clarify the ability of SACTE_2347 to hydrolyze branched mannans (Figure 5). The hydrolysis of **6** resulted in the formation of **5** and **1**. Thus **6** must bind across the −2 to +1 subsites to react to give the observed products but also to satisfy the steric constraints imparted by L1. Reactions that contained **7** yielded **8** and **2**. There was no further breakdown of **8**, an oligomannoside with adjacent branches. This can be attributed to unfavorable steric interactions of the 6^1^-galactosyl branch with L1, which interferes with productive binding leading to catalysis.

### Relative activities of the SACTE_2347 variants

It has been suggested that GH26 enzymes, which lack CBM domains, can preferentially hydrolyze soluble substrates, whereas GH5 enzymes, which possess CBM domains, have preferential utility in hydrolysis of insoluble substrates [27]. The different variants of SACTE_2347 had similar *k*_cat_/*K*_M_ values (Table 3), although SACTE_2347_FL, which uniquely contains the CBM2 domain, had a modestly improved *k*_cat_/*K*_M_ when reacted with pure mannan. The observed kinetic parameters for SACTE_2347 were comparable to that of TfManA, the closest structural homolog [32]. Furthermore, although SACTE_2347_FL was the largest protein studied, it also showed the best specific activity with IL-pine, demonstrating the utility of the attached CBM2 domain.

### Mannanase in Symbiotic Communities

SirexAA-E was isolated from the evolutionarily specialized *Sirex*/fungus/bacteria community [11]. The fungal and bacterial members of this community make important contributions to the invasive nature of the insect infestation through the deconstructive activities of their secreted enzymes. Since pinewood has a large fraction of mannan, it is perhaps not surprising that SACTE_2347 is the fourth most abundant protein secreted by SirexAA-E. It is somewhat surprising, however, that SirexAA-E does not use mannan as a growth substrate, suggesting other members of the community that use these compounds may be cross-fed by the action of this enzyme. Similar considerations apply to the non-reactive branched galactomannans **5** and **8**, which are likely further processed by other enzymes from the microbial community.

Proteolytic processing of cellulases and xylanases has been studied [21], [33]–[35]. For example, xylanase from *Streptomyces halstedii* JM8 was proteolytically cleaved into two different forms, Xys1L (45 kDa) and Xys1S (35 kDa) [21], [36]. Both forms were reported to be similarly active on soluble and insoluble oat spelt xylan and birchwood xylan. Similarly, cellulase (Avicellase) from *Streptomyces reticuli* was proteolytically processed, and the full-length enzyme was shown to be more active than processed enzyme with cellulose polymers (Avicel, carboxymethylcellulose, and hydroxyethylcellulose), whereas the 42-kDa variant had higher activity than the full-length enzyme with para-nitrophenylcellobioside [35]. Proteolytically processing of a mannosidase in the Lilly flower plant has been reported [37], but no other studies on the consequences of proteolysis of mannanases have been reported.

The external environment of the larval feeding tunnel, where SirexAA-E lives, is a harsh environment where H_2_O_2_, metal-containing oxidative enzymes, and numerous proteases are present. Moreover, exposed lignin provides a surface where enzymes might be non-specifically adsorbed and inactivated. Our mass spectral results indicate that proteolysis of SACTE_2347 occurs at specific inter-domain positions. Once formed, the proteolyzed variants are relatively stable. Proteolytic processing of SACTE_2347 in the larval tunnel may overcome non-specific adsorption, particularly if the attachment occurs through the CBM domain. Interestingly, all three forms of SACTE_2347 had equivalent *k*_cat_/*K*_M_ and specific activity for reaction with locust bean gum, a galactomannan representative of the mannan fraction present in pine. These results suggest each of the naturally proteolyzed forms of SACTE_2347 present in the SirexAA-E secretome play a role in biomass processing in the larval feeding tunnel.

## Materials and Methods

### Genomic Analyses

The SirexAA-E genome can be found in the NCBI databank (GenBank: CP002993.1). The domain structure of SACTE_2347 was annotated by the US DOE Joint Genome Institute as part of their genome sequencing efforts [38]. Microarray and proteomic analyses were given in a previous study [11], and these data were deposited in the Gene Expression Omnibus (GEO) database (GSE31748), and in the EBML-EBI Proteomics Identifications database (24850), respectively.

### Protein Sequence Alignments

The SACTE_2347 sequence can be found in the Uniprot database (G2NHM6, [38]). Other protein sequences described in this study were obtained from Pfam (http://pfam.sanger.ac.uk/ [39]) or CAZy (http://www.cazy.org/ [25]). Biochemical characteristics of GH5 subfamily 8 (101 members) were obtained from CAZy. Sequence alignments and phylogenic trees were generated using MegAlign (DNAstar, WI) with 1000 cycles of bootstrap.

### Preparation of SACTE_2347 β-mannanase

Preparation of the SirexAA-E secretome and fractionation of enzymes were described previously [11]. Briefly, SirexAA-E was grown for 7 days at 30°C in M63 minimal medium containing Sigmacell-20 (Sigma-Aldrich, MO) as a sole carbon source. The culture supernatant was collected by centrifugation for 20 min at 4,200×*g* at 4°C and then filter-sterilized using a 0.20 μm filter (Polyethersulfon, Sartorius stedim, Goettingen, Germany). The recovered protein was concentrated to ∼10 mg/mL by centrifugal ultrafiltration (VIVASPIN 20, Sartorius stedim, Goettingen, Germany). The secretome was fractionated by anion exchange chromatography using a 1.6 cm dia ×10.0 cm bed height Mono-Q column (GE HealthCare, Piscataway, NJ) equilibrated in 20 mM Tris-HCl, pH 7.0. The bound protein was eluted in a 100 mL linear gradient of buffer changed from 0 to 1 M NaCl. Fractions containing mannanase activity were identified by assay with β-1,4 D-mannan and visual inspection of SDS-PAGE gels.

### N-terminal Peptide Sequencing

Samples from the anion exchange chromatography that contained mannanase activity were transferred to a PVDF membrane using a semidry blotting system (Bio-Rad laboratories, Hercules, CA). Three polypeptides were excised from the PVDF membrane and submitted for N-terminal sequencing at the Protein Facility Iowa State University Office of Biotechnology (Ames, Iowa, USA).

### Mass spectrometry for protein sequence determination

Protein samples were precipitated in 80% acetone, washed once in ice-cold methanol, solubilized in neat formic acid and then diluted 10-fold in 50∶50 methanol:water for analysis. The protein sample (0.5 μL) was deposited onto an Opti-TOF 384 well plate (Applied Biosystems, Foster City, CA) and suspended with 0.4 μL of 10 mg/mL solution of α-cyano-4-hydroxycinnamic acid dissolved in acetonitrile/H_2_O/TFA (70/30/0.1). Mass spectra were acquired on a 4800 MALDI TOF-TOF mass spectrometer (Applied Biosystems, Foster City, CA) by scanning a 20,000–160,000 Da mass range. 1000 shots were acquired from 25 randomized regions of the sample spot at 5,300 intensity and 0.9 V detector voltage multiplier with an OptiBeam on-axis Nd:YAG laser with 200 Hz firing rate and 3 to 7 ns pulse width in positive linear high mass mode. External calibration of mass accuracy was performed with bovine serum albumin, where the accuracy of experimental mass of 33222 [M+2H]^+^ and 66446 [M+2H]^+^, compared to theoretical mass of 33217 [M+2H]^+^ and 66431 [M+2H]^+^ were confirmed, respectively.

Tryptic digestion of polypeptides transferred to PVDF membranes and mass spectral analyses of released peptides were carried out in the Mass Spectrometry Facility (Biotechnology Center, University of Wisconsin-Madison). The tryptic peptides were analyzed by nanoLC-MS/MS using an Agilent 1100 nanoflow system (Agilent, Palo Alto, CA) connected to a hybrid linear ion trap-orbitrap mass spectrometer (LTQ-Orbitrap, Thermo Fisher Scientific, Germany) equipped with a nanoelectrospray ion source. Raw MS/MS data were converted to the mgf file format using Trans Proteomic Pipeline (Seattle Proteome Center, WA). Resulting mgf files were used to search a user-defined amino acid sequence database with an in-house Mascot search engine 2.2.07 (Matrix Science, UK) that defined Cys carbamidomethylation as a fixed modification and Met oxidation and Asn/Gln deamidation as variable modifications. The peptide mass tolerance was set at 10 ppm and the fragment mass tolerance was set at 0.8 Da. Protein annotations and the probable significance of the identifications were carried out using Scaffold (version 3.6.1, Proteome Software Inc., OR).

### Recombinant SACTE_2347

Three forms of the SACTE_2347 gene were amplified from SirexAA-E genomic DNA by a two-step PCR method [40], [41]. The same forward primer was used for all constructs: 5′-AACCTGTACTTCCAGTCCGCCGCCGGCCTCCACAT-3′. Different reverse primers were used to clone sequences encoding 34 kDa (5′-GCTCGAATTCGTTTAAACTATCACGTCGGGGTGCCGGGTG-3′), 42 kDa (5′-GCTCGAATTCGTTTAAACTATCACCCGGGCACGCTGC-3′), and full-length variants (5′-GCTCGAATTCGTTTAAACTATCAGGCCGCGGTGCAGG-3′) of SACTE_2347. The amplified PCR products were digested using *Sgf*I and *Pme*I (Promega, Madison, WI) followed by ligation into pVP67K. The ligated plasmids were transformed into *Escherichia coli* BL21 (DE3) and plated onto Luria Bertani medium supplemented with kanamycin (50 μg/mL) and chloramphenicol (34 μg/mL). Plasmids containing the proper inserts were identified from successful transformants by nucleotide sequencing using the following universal forward (5′-GGTTGCGATCGCCGAAAACCTGTACTTCCAG-3′) and reverse (5′-GTGTGAGCTCGAATTCGTTTAAACC-3′) primers (Biotechnology Center, University of Wisconsin-Madison).

*E. coli* BL21 (DE3) transformed with sequence-verified plasmids encoding the different SACTE_2347 constructs were grown in 2 mL of non-inducing medium for 12 h at room temperature [42]. For scale-up, 2 mL aliquots were transferred into 200 mL of non-inducing medium and grown for 12 h at room temperature. The 25 mL culture was then transferred into 1 L of auto-induction medium containing kanamycin (50 μg/mL) and chloramphenicol (34 μg/mL), and incubated for 25 h at 25°C. Cells were harvested by centrifugation at 5,000×*g* for 15 min, and the cell paste was suspended at ∼1∶1 (w/v) in 20 mM Tris HCl, pH 7.0, supplemented with a protease inhibitor cocktail containing 1 μM E-64 (Sigma-Aldrich, MO), 0.5 mM benzamidine (Calbiochem, Spring Valley, CA), and 1 mM EDTA. The cell suspension was placed in an ice bath and sonicated for 10 min with a duty cycle of 15 s on and 15 s off. The sonicated cell suspension was centrifuged at 20,000×*g* for 60 min and the supernatant was loaded onto a 1.6 cm dia ×2.5 cm bed height HisTrap HP affinity column (GE Healthcare, Piscataway, NJ) equilibrated in 10 mM MOPS, pH 7.0, containing 500 mM NaCl. The column was washed with 10 volumes of equilibration buffer, and the bound protein was eluted with a linear 100 mL gradient prepared from equilibration buffer and equilibration buffer supplemented with 0.5 M imidazole). Tobacco etch virus protease was used to remove the His tag from all SACTE_2347 preparations that were used for biochemical studies. Briefly, 0.04 mg of purified His-tagged TEV protease [43] was mixed with 1 mg of SACTE_2347, and incubated for 12 h at 4°C with mild agitation. Subtractive IMAC purification was carried out using a 1.6 cm dia ×2.5 cm bed height HisTrap HP affinity column. Fractions containing SACTE_2347 were collected in the flow-through from the HisTrap column. The purity of SACTE_2347 preparations was estimated by SDS-PAGE, and the protein concentration was estimated by BCA assay (Bio-Rad, Hercules, CA).

### Enzyme Substrates

Substrates for enzyme assays were from Sigma-Aldrich except as indicated. Fluorogenic substrates were 4-methylumbelliferyl-β-D-glucopyranoside (MUG), 4-methylumbelliferyl-β-D-cellobioside (MUC), 4-methylumbelliferyl-β-D-xylopyranoside (MUX), 4-methylumbelliferyl-β-D-mannopyranoside (MUM), 4-methylumbelliferyl-β-D-galactopyranoside (MUGal), 4-methylumbelliferyl-β-D-fucoside (MUF) and 4-methylumbelliferyl-β-D-acetate (MUA). Insoluble polysaccharides were Sigmacell-20, birchwood xylan, acetylated glucomannan, locust bean gum (∼3.5 mannose per galactose), galactan, mannose, and mannobiose. β-1,4 D-mannan, mannotriose, mannotetraose, 6^1^-α-D-galactosyl-mannotriose, 6^1^-α-D-galactosyl-mannobiose, and 6^3^,6^4^-α-D-galactosyl-mannopentaose were from Megazyme (Wicklow, Ireland). Ammonia fiber expansion-treated cornstover (AFEX-CS) and ammonia fiber expansion-treated switchgrass (AFEX-SG) were provided by the Great Lakes Bioenergy Research Center [23]. Ionic liquid-treated samples of mixed biomass (IL-MB) and pine wood (IL-pine) was the generous gift of the Joint Bioenergy Institute (Emeryville, CA).

### Zymogram Assays

Fractions from anion exchange chromatography containing SACTE_2347 were electrophoresed under non-denaturing conditions in a 12% PAGE gel containing 0.1% β-1,4 D-mannan. After the electrophoresis was complete, the gel was incubated for 10 min at 25°C to allow mannan hydrolysis to proceed. Afterwards, the gel was stained in 0.1% Congo Red for 10 min and washed in 1 M NaCl, at room temperature. To identify the location of protein, a comparable 12% PAGE gel lacking mannan was prepared using the same voltage and time and stained with Coomassie Blue. Both gel images were captured using the Gel Doc EZ imager (Bio-Rad laboratories, Hercules, CA)

### Enzyme Assays

For assays using the fluorogenic substrates, 0.2 mM samples of the substrate were prepared immediately before use in 0.1 M sodium phosphate, pH 8.0. In the enzyme assay, 1 μg of enzyme was mixed with 25 μL of the 0.2 mM fluorogenic substrate and 0.1 M sodium phosphate, pH 8.0, to give a total reaction volume of 100 μL. The reaction was carried out for 30 min at 37°C. The fluorescence measurement was performed using excitation and emission detection at 360 nm and 460 nm, respectively. Fluorescence measurements were corrected for a minor (∼1%) non-enzymatic hydrolysis of the MU substrates during the time course of the reaction.

Steady-state kinetics studies with purified polysaccharides were carried out in 50 μL of 50 mM phosphate, pH 6.0, containing 20 μg/mL of enzyme. Steady-state kinetics measurements with biomass substrates were carried out in 100 μL of 50 mM phosphate, pH 6.0, with 100 μg/mL of enzyme. The amounts of insoluble substrate and biomass were varied to achieve the following weight loadings (mg/mL): 0, 0.5, 1.0, 2.5, 5.0 and 10.0. To determine the amount of soluble reducing sugar released, DNS assays were used as previously described [44]. For this work, a unit of enzyme activity (U) is defined as the release of 1 μmol of reducing sugar per minute from mannan (1,4- β -D-mannan, Megazyme) in 50 mM phosphate, pH 6.0 at 40°C. Results were analyzed using Prism 6.0 (GraphPad, La Jolla, CA).

The identities and proportions of soluble sugar oligomers obtained from enzymatic hydrolysis reactions were determined using an HPLC equipped with an RID-10A refractive index detector (Shimadzu Scientific Instruments, Columbia, MD) and a Rezex RPM-oligosaccharide column (Phenomenex, Torrance, CA). Distilled and deionized water was used as the mobile phase with a flow rate of 0.3 mL min^−1^ at 85°C. Pure d-mannose, mannobiose, mannotriose, mannotetraose, 6^1^-α- D-galactosyl-mannobiose, 6^1^-α-D-galactosyl-mannotriose, and 6^3^,6^4^-α-D-galactosyl-mannopentaose were used as controls to determine retention times and to produce calibration curves for refractive index response as a function of concentration. These materials were also used as substrates in some enzyme assays.

The incorporation of ^18^O from ^18^O-enriched water (97% ^18^O, Cambridge Isotope Laboratories Inc, MA) into mannose (**1**) and mannobiose (**2**) during the reaction with mannotriose (**3**, Figure 5) was determined by the ESI-TOF mass spectrometry in negative ion mode (Agilent ESI-TOF model number G1969A). Samples were prepared by incubating 10 mg/mL of SACTE_2347_34kDa with 10 μM **3** in reaction mixture containing ∼10% enrichment of ^18^O for 30 min. A 2.5 μL portion of the sample was directly injected and delivered to the electrospray source using a 1∶1 mixture of acetonitrile:water at 40 μL/min flow rate. Internal calibration was achieved by supplying Agilent calibrant mix at 10 μL/min to the second electrospray needle in the dual-spray source. The percentage incorporation of ^18^O into either **1** or **2** was calculated from the ratio of the intensity of the mass spectral signals arising from the labeled and unlabeled products. Parallel control reactions showed no ^18^O incorporation into products in the absence of enzyme.

### Insoluble Polysaccharide Binding Assays

Pull down assays were carried out using Sigmacell-20, β-1,4 D-mannan, xylan, AFEX-CS, AFEX-SG, IL-SG, IL-MB, IL-pine, and lignin isolated from pine wood and poplar [23], [24], and two synthetic lignin compounds (G-DHP and G/S-DHP) [24]. A 10 μg sample of enzyme was incubated with 1 mg of substrate in 50 mM phosphate buffer, pH 7.0, for 1 h at 4°C, and then the sample was centrifuged at 12,000×*g* for 5 min at 4°C. The supernatant (containing unbound enzyme) and pellet (containing bound enzyme) were separated and normalized amounts of the two fractions were dissolved in denaturing buffer and separated using 4–20% gradient SDS-PAGE. Incubations were performed without substrate as a control for the possibility that the enzyme alone was precipitated during the incubation period, and none was observed.

### X-ray Structure Determination

Initial crystallization screening was carried out using a Mosquito nanoliter liquid handling robot (TTP LabTech, Cambridge, MA) at 277 and 293 K. Crystals were observed in the Joint Center for Structural Genomics HT Screen (Hampton Research, Aliso Viejo, CA) at 293 K. Preparations of SACTE_2347_34kDa and a derivative where the His tag was removed by treatment with TEV protease [43] were placed into crystallization trials, and only the enzyme containing the His tag yielded crystals. Crystals that were used to solve the SACTE_2347_34kDa structure were grown by hanging-drop vapor-diffusion by mixing 1 μL of 20 mg/mL protein solution with an equal volume of 100 mM Bis-Tris, pH 6.0, containing 21% (w/v) PEG 3350 and 200 mM magnesium chloride. SACTE_2347_34kDa crystals were cryoprotected with Fomblin 2500 (Sigma-Aldrich) and directly frozen in liquid N_2_.

Diffraction data were collected at Life Sciences-Collaborative Access Team beamline 21-ID-G at the Advanced Photon Source, Argonne National Laboratory. Diffraction images were indexed, integrated, and scaled using HKL2000 [45]. The structure of SACTE_2347_34kDa was solved by molecular replacement with PHASER [46] using ManA from *Thermomonospora fusca* as the initial model (PDB ID 1BQC, [17]). The resulting electron density map was of high quality and the SACTE_2347_34kDa structure was determined with iterative rounds of model building in Coot [47] and refinement in PHENIX [48]. Due to the high resolution of the SACTE_2347_34kDa structure, all non-hydrogen atoms were refined using anisotropic atomic displacement factors. Structure images were created using PyMol [49].

Coordinates and structure factors have been deposited in the Protein Data Bank (PBD) as 4FK9.

## Supporting Information

Figure S1 **Protein sequence of SACTE_2347 mannanase with detected peptide by mass spectrometry.** Domain structure (A) and a protein sequence with peptides described in Table 1 (B) are shown.
(DOCX)

Figure S2 **Sequence alignment of SACTE_2347 mannanase and three other mannanases.** SACTE_2347 mannanase (PDB 4FK9), *T. fusca* mannanase (PDB 1BQC [1]), Bacillus sp. JAMB-602 (PDB 1WKY), and Bacillus sp. N16-5 mannanase (PDB 2WHJ [2]) were aligned with the secondary structure elements annotated, alpha helix (yellow ribbon) and beta sheet (filled light blue arrow). Conserved eight residues Arg100, His136, Asn177, His244, Tyr246, and Trp303 (green box), and Glu178 and Glu273 (orange box), the catalytic acid/base and nucleophile are shown.
(DOCX)

Figure S3 **Optimal reaction conditions of SACTE_2347 mannanase.** pH (A) and temperature (B) profiles for reaction of SACTE_2347 and thermal stability. Mannan hydrolysis was measured by the DNS assay. The maximum activity was observed between pH 6 and 7 (A) and 30 to 40°C (B). The thermal stability of SACTE_2347_34kDa (circle), SACTE_2347_42kDa (square) and SACTE_2347_FL (triangle) are shown. The dashed line indicates 50% relative activity.
(DOCX)

Table S1 **RMSD of GH5 enzymes when unbound and bound structures are available.**
(DOCX)

## References

[1] Petkowicz CLD, Reicher F, Chanzy H, Taravel FR, Vuong R (2001) Linear mannan in the endosperm of *Schizolobium amazonicum* . Carbohydrate Polymers 44: 107–112.

[2] Teleman A, Nordstrom M, Tenkanen M, Jacobs A, Dahlman O (2003) Isolation and characterization of O-acetylated glucomannans from aspen and birch wood. Carbohydr Res 338: 525–534. 12668108 10.1016/s0008-6215(02)00491-3

[3] Liepman AH, Nairn CJ, Willats WGT, Sorensen I, Roberts AW, et al (2007) Functional genomic analysis supports conservation of function among cellulose synthase-like a gene family members and suggests diverse roles of mannans in plants. Plant Physiology 143: 1881–1893. 17307900 10.1104/pp.106.093989 PMC1851810

[4] Arcand N, Kluepfel D, Paradis FW, Morosoli R, Shareck F (1993) Beta-mannanase of *Streptomyces lividans* 66: cloning and DNA sequence of the manA gene and characterization of the enzyme. Biochem J 290 (Pt 3): 857–863. 10.1042/bj2900857 PMC1132360 8457214

[5] Malek MA, Berry DR (1995) Isolation and partial characterization of yeast mannan hydrolysing enzymes from bacterial isolates. Microbios 83: 229–241. 8577261

[6] Tamaru Y, Araki T, Amagoi H, Mori H, Morishita T (1995) Purification and characterization of an extracellular beta-1,4-mannanase from a marine bacterium, *Vibrio* sp. strain MA-138. Appl Environ Microbiol 61: 4454–4458. 8534110 10.1128/aem.61.12.4454-4458.1995 PMC167754

[7] Dhawan S, Kaur J (2007) Microbial mannanases: an overview of production and applications. Crit Rev Biotechnol 27: 197–216. 18085462 10.1080/07388550701775919

[8] Moreira LR, Filho EX (2008) An overview of mannan structure and mannan-degrading enzyme systems. Applied microbiology and biotechnology 79: 165–178. 18385995 10.1007/s00253-008-1423-4

[9] Shallom D, Shoham Y (2003) Microbial hemicellulases. Curr Opin Microbiol 6: 219–228. 12831897 10.1016/s1369-5274(03)00056-0

[10] Adams AS, Jordan MS, Adams SM, Suen G, Goodwin LA, et al (2011) Cellulose-degrading bacteria associated with the invasive woodwasp *Sirex noctilio* . The ISME journal 5: 1323–1331. 21368904 10.1038/ismej.2011.14 PMC3146269

[11] Takasuka TE, Book AJ, Lewin GR, Currie CR, Fox BG (2013) Aerobic deconstruction of cellulosic biomass by an insect-associated *Streptomyces* . Sci Rep 3: 1030. 23301151 10.1038/srep01030 PMC3538285

[12] Bianchetti CM, Harmann CH, Takasuka TE, Hura GL, Dyer K, et al (2013) Fusion of dioxygenase and lignin-binding domains in a novel secreted enzyme from cellulolytic *Streptomyces* sp. SirexAA-E. J Biol Chem 288: 18574–18587. 23653358 10.1074/jbc.M113.475848 PMC3689997

[13] Wickner W, Schekman R (2005) Protein translocation across biological membranes. Science 310: 1452–1456. 16322447 10.1126/science.1113752

[14] Wang QP, Tull D, Meinke A, Gilkes NR, Warren RAJ, et al (1993) Glu280 Is the Nucleophile in the Active-Site of *Clostridium thermocellum* Celc, a Family-a Endo-Beta-1,4-Glucanase. Journal of Biological Chemistry 268: 14096–14102. 8100226

[15] Vasella A, Davies GJ, Bohm M (2002) Glycosidase mechanisms. Curr Opin Chem Biol 6: 619–629. 12413546 10.1016/s1367-5931(02)00380-0

[16] White A, Tull D, Johns K, Withers SG, Rose DR (1996) Crystallographic observation of a covalent catalytic intermediate in a beta-glycosidase. Nat Struct Biol 3: 149–154. 8564541 10.1038/nsb0296-149

[17] Hilge M, Gloor SM, Rypniewski W, Sauer O, Heightman TD, et al (1998) High-resolution native and complex structures of thermostable beta-mannanase from *Thermomonospora fusca* - substrate specificity in glycosyl hydrolase family 5. Structure 6: 1433–1444. 9817845 10.1016/s0969-2126(98)00142-7

[18] Davies GJ, Wilson KS, Henrissat B (1997) Nomenclature for sugar-binding subsites in glycosyl hydrolases. Biochem J 321 (Pt2): 557–559. 9020895 10.1042/bj3210557 PMC1218105

[19] Barr BK, Hsieh YL, Ganem B, Wilson DB (1996) Identification of two functionally different classes of exocellulases. Biochemistry 35: 586–592. 8555231 10.1021/bi9520388

[20] Bianchetti CM, Brumm P, Smith RW, Dyer K, Hura GL, et al (2013) Structure, dynamics, and specificity of endoglucanase D from Clostridium cellulovorans. J Mol Biol 425: 4267–4285. 23751954 10.1016/j.jmb.2013.05.030 PMC4039632

[21] Ruiz-Arribas A, Sanchez P, Calvete JJ, Raida M, Fernandez-Abalos JM, et al (1997) Analysis of xysA, a gene from *Streptomyces halstedii* JM8 that encodes a 45-kilodalton modular xylanase, Xys1. Appl Environ Microbiol 63: 2983–2988. 9251186 10.1128/aem.63.8.2983-2988.1997 PMC168597

[22] Xu GY, Ong E, Gilkes NR, Kilburn DG, Muhandiram DR, et al (1995) Solution structure of a cellulose-binding domain from *Cellulomonas fimi* by nuclear magnetic resonance spectroscopy. Biochemistry 34: 6993–7009. 7766609

[23] Li C, Cheng G, Balan V, Kent MS, Ong M, et al (2011) Influence of physico-chemical changes on enzymatic digestibility of ionic liquid and AFEX pretreated corn stover. Bioresour Technol 102: 6928–6936. 21531133 10.1016/j.biortech.2011.04.005

[24] Tobimatsu Y, Elumalai S, Grabber JH, Davidson CL, Pan X, et al (2012) Hydroxycinnamate conjugates as potential monolignol replacements: in vitro lignification and cell wall studies with rosmarinic acid. ChemSusChem 5: 676–686. 22359379 10.1002/cssc.201100573

[25] Aspeborg H, Coutinho PM, Wang Y, Brumer H 3rd, Henrissat B (2012) Evolution, substrate specificity and subfamily classification of glycoside hydrolase family 5 (GH5). BMC Evol Biol 12: 186. 22992189 10.1186/1471-2148-12-186 PMC3526467

[26] Akita M, Takeda N, Hirasawa K, Sakai H, Kawamoto M, et al (2004) Crystallization and preliminary X-ray study of alkaline mannanase from an alkaliphilic Bacillus isolate. Acta Crystallogr D Biol Crystallogr 60: 1490–1492. 15272186 10.1107/S0907444904014313

[27] Hagglund P, Eriksson T, Collen A, Nerinckx W, Claeyssens M, et al (2003) A cellulose-binding module of the *Trichoderma reesei* beta-mannanase Man5A increases the mannan-hydrolysis of complex substrates. J Biotechnol 101: 37–48. 12523968 10.1016/s0168-1656(02)00290-0

[28] Sabini E, Schubert H, Murshudov G, Wilson KS, Siika-Aho M, et al (2000) The three-dimensional structure of a *Trichoderma reesei* beta-mannanase from glycoside hydrolase family 5. Acta Crystallogr D Biol Crystallogr 56: 3–13. 10666621 10.1107/s0907444999013943

[29] Tailford LE, Ducros VM, Flint JE, Roberts SM, Morland C, et al (2009) Understanding how diverse beta-mannanases recognize heterogeneous substrates. Biochemistry 48: 7009–7018. 19441796 10.1021/bi900515d

[30] Zhang Y, Ju J, Peng H, Gao F, Zhou C, et al (2008) Biochemical and structural characterization of the intracellular mannanase AaManA of *Alicyclobacillus acidocaldarius* reveals a novel glycoside hydrolase family belonging to clan GH-A. J Biol Chem 283: 31551–31558. 18755688 10.1074/jbc.M803409200

[31] Gilbert HJ (2010) The biochemistry and structural biology of plant cell wall deconstruction. Plant Physiol 153: 444–455. 20406913 10.1104/pp.110.156646 PMC2879781

[32] Kumagai Y, Kawakami K, Mukaihara T, Kimura M, Hatanaka T (2012) The structural analysis and the role of calcium binding site for thermal stability in mannanase. Biochimie 94: 2783–2790. 23009928 10.1016/j.biochi.2012.09.012

[33] Adham SA, Honrubia P, Diaz M, Fernandez-Abalos JM, Santamaria RI, et al (2001) Expression of the genes coding for the xylanase Xys1 and the cellulase Cel1 from the straw-decomposing *Streptomyces halstedii* JM8 cloned into the amino-acid producer *Brevibacterium lactofermentum* ATCC13869. Arch Microbiol 177: 91–97. 11797049 10.1007/s00203-001-0365-3

[34] Moormann M, Schlochtermeier A, Schrempf H (1993) Biochemical Characterization of a Protease Involved in the Processing of a *Streptomyces reticuli* Cellulase (Avicelase). Appl Environ Microbiol 59: 1573–1578. 16348937 10.1128/aem.59.5.1573-1578.1993 PMC182121

[35] Schlochtermeier A, Walter S, Schroder J, Moorman M, Schrempf H (1992) The gene encoding the cellulase (Avicelase) Cel1 from *Streptomyces reticuli* and analysis of protein domains. Molecular microbiology 6: 3611–3621. 1282194 10.1111/j.1365-2958.1992.tb01797.x

[36] Ruiz-Arribas A, Fernandez-Abalos JM, Sanchez P, Garda AL, Santamaria RI (1995) Overproduction, purification, and biochemical characterization of a xylanase (Xys1) from *Streptomyces halstedii* JM8. Appl Environ Microbiol 61: 2414–2419. 7793962 10.1128/aem.61.6.2414-2419.1995 PMC167513

[37] Ishimizu T, Sasaki A, Okutani S, Maeda M, Yamagishi M, et al (2004) Endo-beta-mannosidase, a plant enzyme acting on N-glycan: purification, molecular cloning, and characterization. J Biol Chem 279: 38555–38562. 15247239 10.1074/jbc.M406886200

[38] UniProt C (2012) Reorganizing the protein space at the Universal Protein Resource (UniProt). Nucleic Acids Res 40: D71–75. 22102590 10.1093/nar/gkr981 PMC3245120

[39] Finn RD, Mistry J, Tate J, Coggill P, Heger A, et al (2010) The Pfam protein families database. Nucleic acids research 38: D211–222. 19920124 10.1093/nar/gkp985 PMC2808889

[40] Blommel PG, Martin PA, Seder KD, Wrobel RL, Fox BG (2009) Flexi vector cloning. Methods Mol Biol 498: 55–73. 18988018 10.1007/978-1-59745-196-3_4 PMC5957505

[41] Takasuka TE, Walker JA, Bergeman LF, Meulen KA, Makino S, et al (2014) Cell-free translation of biofuel enzymes. Methods Mol Biol 1118: 71–95. 24395410 10.1007/978-1-62703-782-2_5 PMC5820533

[42] Blommel PG, Becker KJ, Duvnjak P, Fox BG (2007) Enhanced bacterial protein expression during auto-induction obtained by alteration of lac repressor dosage and medium composition. Biotechnol Prog 23: 585–598. 17506520 10.1021/bp070011x PMC2747370

[43] Blommel PG, Fox BG (2007) A combined approach to improving large-scale production of tobacco etch virus protease. Protein Expr Purif 55: 53–68. 17543538 10.1016/j.pep.2007.04.013 PMC2047602

[44] Miller GL (1959) Use of dinitrosalicylic acid reagent for determination of reducing sugar. Anal Chem 31: 426–428.

[45] Otwinowski Z, and Minor W (1997) Processing of x-ray diffraction data collected in oscillation mode. Macromolecular Crystallography Part A 307–326. 10.1016/S0076-6879(97)76066-X 27754618

[46] McCoy AJ, Grosse-Kunstleve RW, Adams PD, Winn MD, Storoni LC, et al (2007) Phaser crystallographic software. J Appl Crystallogr 40: 658–674. 19461840 10.1107/S0021889807021206 PMC2483472

[47] Emsley P, Cowtan K (2004) Coot: model-building tools for molecular graphics. Acta Crystallogr D Biol Crystallogr 60: 2126–2132. 15572765 10.1107/S0907444904019158

[48] Adams PD, Afonine PV, Bunkoczi G, Chen VB, Davis IW, et al (2010) PHENIX: a comprehensive Python-based system for macromolecular structure solution. Acta Crystallogr D Biol Crystallogr 66: 213–221. 20124702 10.1107/S0907444909052925 PMC2815670

[49] Delano WL (2002) The PyMOL Molecular Graphics System. Delano Scientific LLC, San Carlos, CA, USA.

