# Supplementary material for: Biochemical Properties and Atomic Resolution Structure of a Proteolytically Processed β-Mannanase from Cellulolytic Streptomyces sp. SirexAA-E
Source: PLoS One. 2014 Apr 7;9(4):e94166. doi: 10.1371/journal.pone.0094166 (PMC3978015; doi:10.1371/journal.pone.0094166)
Supplement: Figure S1 — Protein sequence of SACTE_2347 mannanase with detected peptide by mass spectrometry. Domain structure (A) and a protein sequence with peptides described in Table 1 (B) are shown. (DOCX) [file pone.0094166.s001.docx]

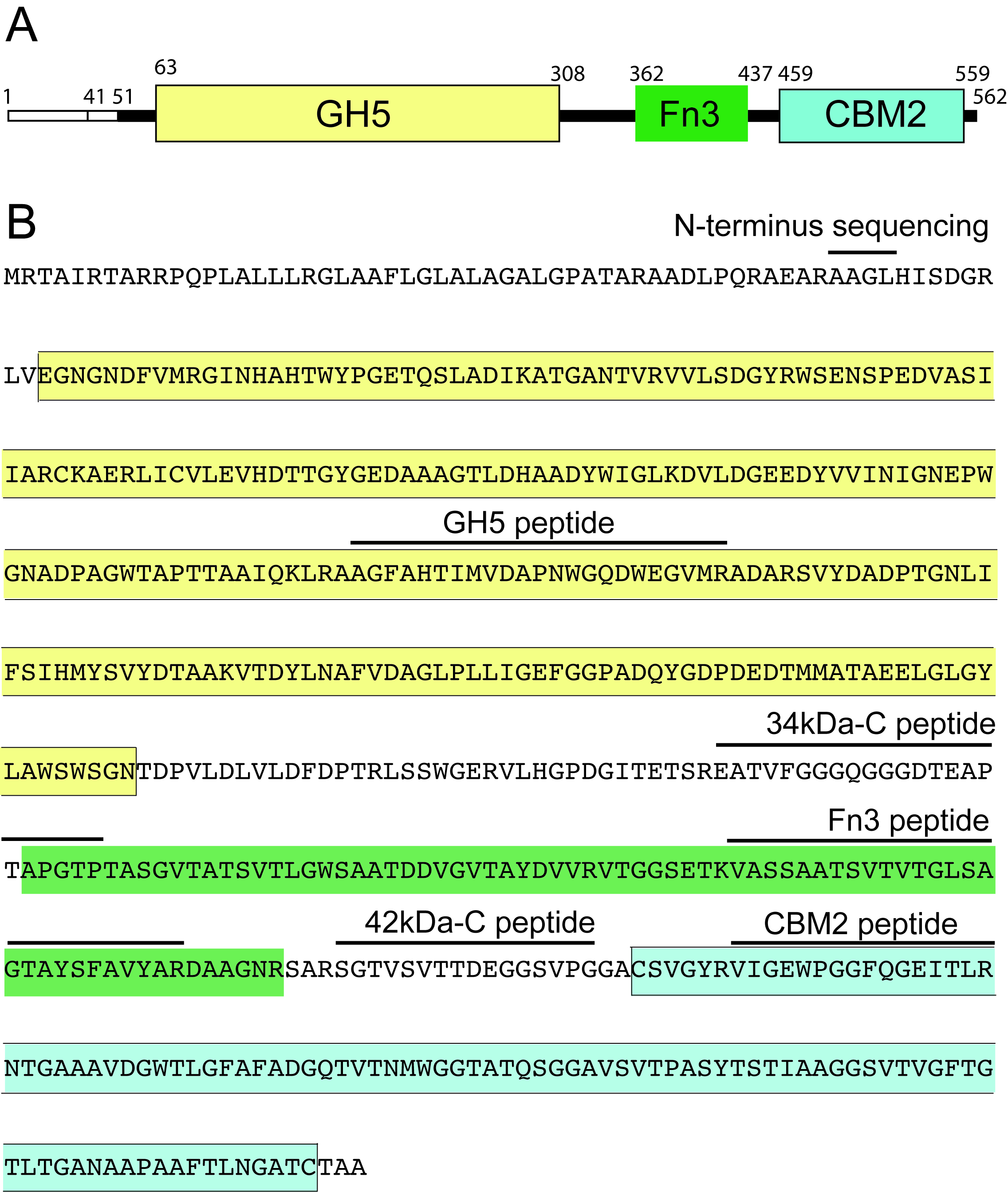


**Figure S1. Protein sequence of SACTE_2347 mannanase annotated with peptide sequences identified by mass spectrometry.** The domain structure of SACTE_2347 (A) and the polypeptide sequence annotated with the positions of the peptides described in Table 1 (B) are shown.
