# Supplementary material for: Biochemical Properties and Atomic Resolution Structure of a Proteolytically Processed β-Mannanase from Cellulolytic Streptomyces sp. SirexAA-E
Source: PLoS One. 2014 Apr 7;9(4):e94166. doi: 10.1371/journal.pone.0094166 (PMC3978015; doi:10.1371/journal.pone.0094166)
Supplement: Figure S2 — Sequence alignment of SACTE_2347 mannanase and three other mannanases. SACTE_2347 mannanase (PDB 4FK9), T. fusca mannanase (PDB 1BQC [1]), Bacillus sp. JAMB-602 (PDB 1WKY), and Bacillus sp. N16-5 mannanase (PDB 2WHJ [2]) were aligned with the secondary structure elements annotated, alpha helix (yellow ribbon) and beta sheet (filled light blue arrow). Conserved eight residues Arg100, His136, Asn177, His244, Tyr246, and Trp303 (green box), and Glu178 and Glu273 (orange box), the catalytic acid/base and nucleophile are shown. (DOCX) [file pone.0094166.s002.docx]

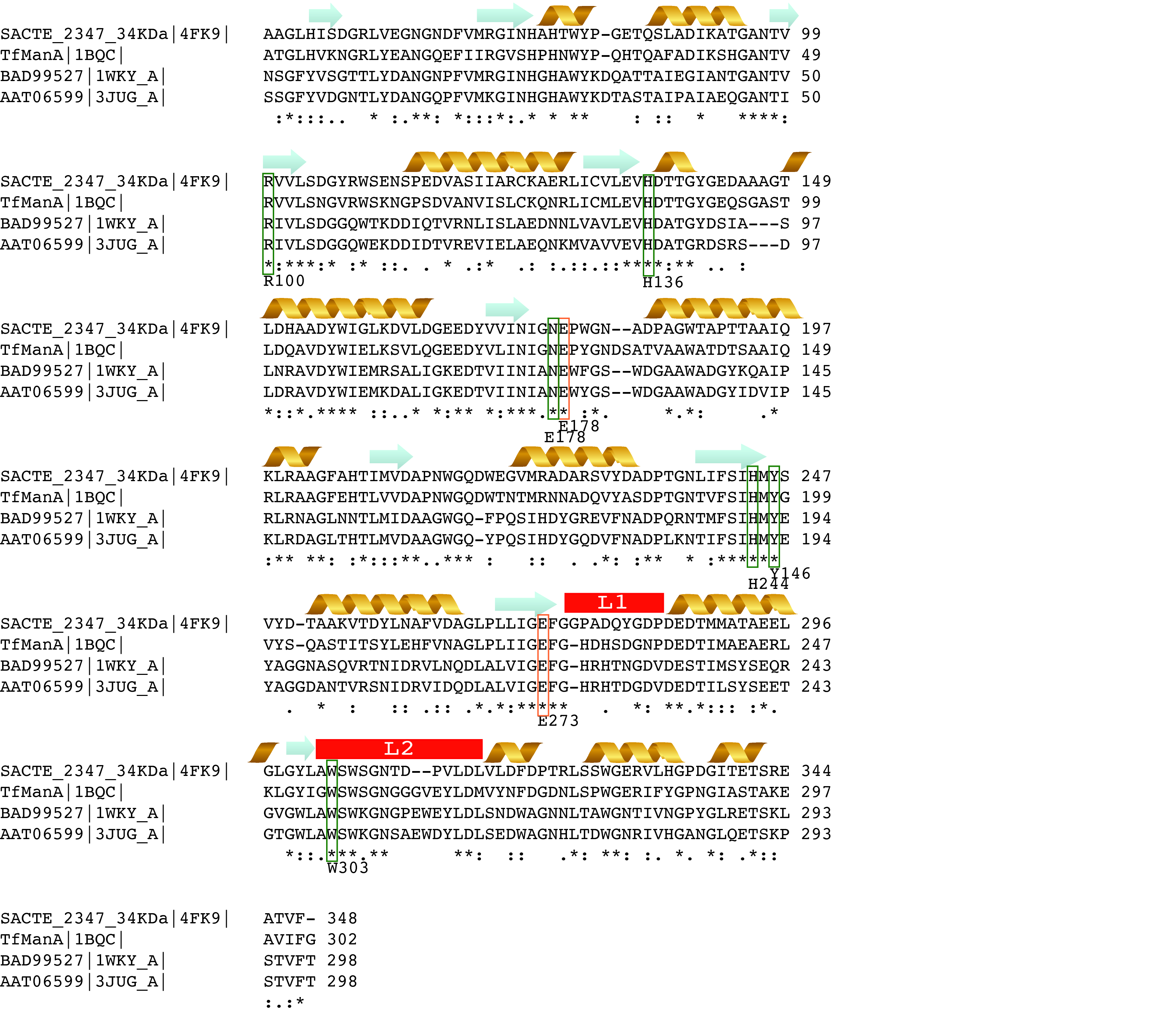


**Figure S2. Alignment of SACTE_2347 mannanase primary sequence with those of three other mannanases.** SACTE_2347 mannanase (PDB 4FK9), *T. fusca* mannanase (PDB 1BQC [1]), *Bacillus* sp. JAMB-602 (PDB 1WKY), and *Bacillus* sp. N16-5 mannanase (PDB 2WHJ [2]) were aligned with the secondary structure elements annotated. Alpha helix (*yellow* ribbon) and beta sheet (filled light *blue* arrow) are shown. Conserved eight residues Arg100, His136, Asn177, His244, Tyr246, and Trp303 (*green* box), and Glu178 and Glu273 (*orange* box), the catalytic acid/base and nucleophile are shown.

**References**

1 Hilge M, Gloor SM, Rypniewski W, Sauer O, Heightman TD, et al. (1998) High-resolution native and complex structures of thermostable beta-mannanase from *Thermomonospora fusca* - substrate specificity in glycosyl hydrolase family 5. Structure 6: 1433-1444.

2 Tailford LE, Ducros VM, Flint JE, Roberts SM, Morland C, et al. (2009) Understanding how diverse beta-mannanases recognize heterogeneous substrates. Biochemistry 48: 7009-7018.
