# Supplementary material for: Biochemical Properties and Atomic Resolution Structure of a Proteolytically Processed β-Mannanase from Cellulolytic Streptomyces sp. SirexAA-E
Source: PLoS One. 2014 Apr 7;9(4):e94166. doi: 10.1371/journal.pone.0094166 (PMC3978015; doi:10.1371/journal.pone.0094166)
Supplement: Figure S3 — Optimal reaction conditions of SACTE_2347 mannanase. pH (A) and temperature (B) profiles for reaction of SACTE_2347 and thermal stability. Mannan hydrolysis was measured by the DNS assay. The maximum activity was observed between pH 6 and 7 (A) and 30 to 40°C (B). The thermal stability of SACTE_2347_34kDa (circle), SACTE_2347_42kDa (square) and SACTE_2347_FL (triangle) are shown. The dashed line indicates 50% relative activity. (DOCX) [file pone.0094166.s003.docx]

Optimal reaction conditions

In short time assays (15 min) using β-1,4 D-mannan as the substrate, SACTE_2347_FL had a temperature optimum for catalytic turnover of ~40 ˚C and retained greater than 50% activity at 55 ˚C (Figure S1A). The pH optimum for catalytic turnover was ~6.5 and the enzyme retained greater than 50% activity in the pH range of 4.5 to 8 (Figure S1B). Similar temperature optima and pH ranges were obtained for the other two SACTE_2347 variants (data not shown). Additionally, the thermal stability was tested (Figure S1C) after 2 h of incubation. SACTE_2347_34kDa and SACTE_2347_42kDa retained 50% activity after incubation at 80 ˚C. In contrast, SACTE_2347_FL was considerably less stable, and only retained 50% activity after incubation at 60 ˚C.


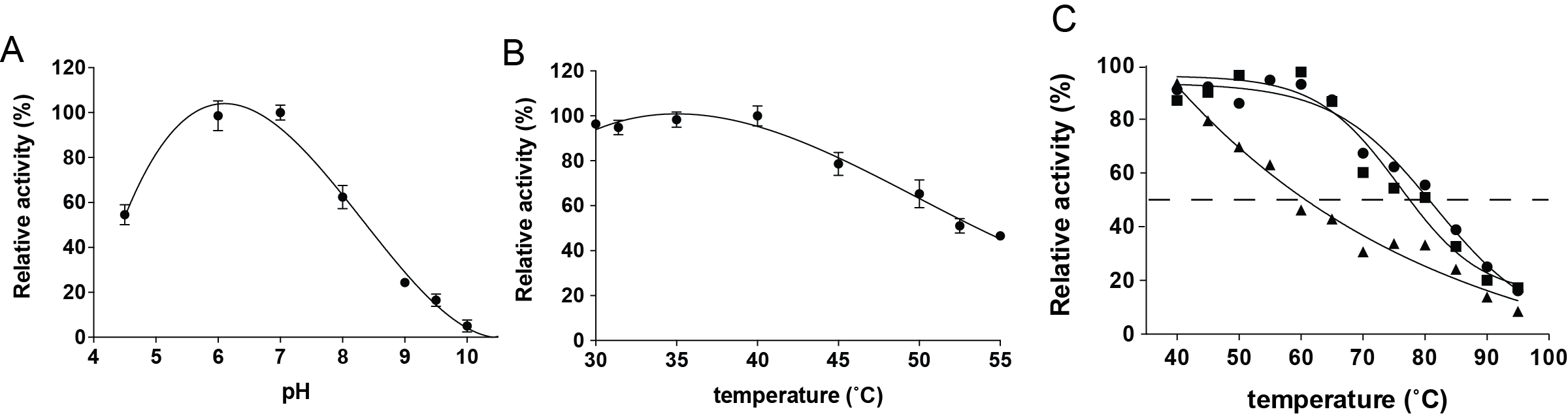


**Figure S3. Optimal reaction conditions of SACTE_2347 mannanase.** pH (A) and temperature (B) profiles for reaction of SACTE_2347 and thermal stability. Mannan hydrolysis was measured by the DNS assay. The maximum activity was observed between pH 6 and 7 (A) and 30 to 40 ˚C (B). The thermal stability of SACTE_2347_34kDa (circle), SACTE_2347_42kDa (square) and SACTE_2347_FL (triangle) are shown. The dashed line indicates 50% relative activity.

**Materials and Methods**

Thermal Stability of SACTE_2347

Samples of each SACTE_2347 variant (1 mg/mL) were incubated in sealed vials containing 50 mM phosphate, pH 6.0, for 2 h at 40, 50, 60, 70, 80, and 90 ˚C. The activity remaining after incubation at the indicated temperatures was determined by reaction with β-1,4 d-mannan at 40 ˚C for 15 min. Reducing sugars were detected by DNS assay.
