# Supplementary material for: Biochemical Properties and Atomic Resolution Structure of a Proteolytically Processed β-Mannanase from Cellulolytic Streptomyces sp. SirexAA-E
Source: PLoS One. 2014 Apr 7;9(4):e94166. doi: 10.1371/journal.pone.0094166 (PMC3978015; doi:10.1371/journal.pone.0094166)
Supplement: Table S1 — RMSD of GH5 enzymes when unbound and bound structures are available. (DOCX) [file pone.0094166.s004.docx]

Structure alignment with other mannanase

Although one monomer was present in the asymmetric unit, the His tag from a crystallographically related monomer was bound in the SACTE_2347 active site. The His tag-free form of the enzyme did not crystallize under any condition tested and attempts to obtain a substrate-bound structure through either soaking or co-crystallization were likewise not successful. Contiguous and interpretable electron density was observed for the His tag, an intervening linker representing the FlexiVector AIA sequence [[1](#_ENREF_1)], the tobacco etch virus protease ENLYFQS recognition site and residues His35-Phe348 from the SACTE_2347 GH5 domain. No electron density, presumably resulting from intrinsic disorder, was observed for residues 31-34 at the N-terminus of the protein and 349-367 at the C-terminus. Both of these disordered regions are on the opposite side of the protein from the active site.

To date, there are 19 GH5 enzymes whose structures have been solved in both unliganded and ligand-bound forms. Alignments between the unliganded and ligand-bound structures display RMSDs from 0.32 Å to 0.1 Å (See Table S1), indicating that no significant movements are required among the GH5 members to bind ligands. Furthermore, CelA from *Piromyces rhizinflatus* 2301 was solved with a His tag from a crystallographically related monomer bound in the active site, as we have observed with SACTE_2347, and also in complex with a potential product inhibitor, cellotriose [[2](#_ENREF_2)]. Alignment of the two CelA structures showed that there is no significant difference between the His tag and cellotriose bound structures (RMSD = 0.24 Å). Thus we conclude that the positions of the residues and loops that line the substrate binding cleft of GH5 enzymes in general, and SACTE_2347_34kDa in specific, will not be changed by the presence of the His tag or other bound ligands.

SACTE_2347_34kDa adopts a (β/α)_8_-barrel fold similar to other structurally determined GH5 mannanases, including β-mannanase from *Thermomonospora fusca*, designated here as TfManA, (PDB ID 1BQC, 0.34 Å rmsd, 74% sequence coverage and 63% sequence identity [[3](#_ENREF_3)]), a two-domain mannanase/domain of unknown function from *Bacillus* sp. strain JAMB-602 (PDB ID 1WKY, 1.3 Å rmsd with the GH5 domain, 56% sequence coverage and 44% sequence identity [[4](#_ENREF_4)]), and β-1,4-mannanase from *Bacillus* sp. N16-5 (PDB ID 3JUG, 1.4 Å rmsd, 59% sequence coverage and 41% sequence identity [[5](#_ENREF_5)]). When compared to the GH5 domain in SACTE_2347, the closest biochemically characterized enzyme is from thermophilic *Streptomyces thermolilacinu* (BAK26781, [[6](#_ENREF_6)]), which has 97% sequence coverage and 52% sequence identity. In addition to these mannanases, SACTE_2347_34kDa shares structurally similarity to distantly related GH5 cellulases [[7-9](#_ENREF_7)].

Table S1. RMSD of GH5 enzymes when unbound and bound structures are available.

| Enzyme name | Source organism | Unbound PDB | Bound PDB | Bound substrate | RMSD (Å) |
| --- | --- | --- | --- | --- | --- |
| EglB | *Pyrococcus horikoshii* OT3 | 2ZUM [[10](#_ENREF_10)] | 3QHN [[11](#_ENREF_11)] | Cellotetraose | 0.31 |
| Cel5A | *Acidothermus cellulolyticus* ATCC 43068 | 1VRX [[12](#_ENREF_12)] | 1ECE [[13](#_ENREF_13)] | Cellotetraose | 0.22 |
| Man5A | *Bacillus agaradhaerens* | 2WHJ [[14](#_ENREF_14)] | 2WHL [[14](#_ENREF_14)] | Mannobiose | 0.22 |
| Egl-K | *Bacillus* sp. KSM-635 | 1G01 [[15](#_ENREF_15)] | 1G0C [[15](#_ENREF_15)] | Cellobiose | 0.12 |
| Man5A | *Cellvibrio mixtus* NCIMB 8633 | 1UUQ [[16](#_ENREF_16)] | 1UZ4 [[17](#_ENREF_17)] | Isofagomine lactam | 0.14 |
| EngD | *Clostridium cellulovorans* ATCC 35269 | 3NDY [[7](#_ENREF_7)] | 3NDZ [[7](#_ENREF_7)] | Cellotriose | 0.18 |
| Cel5B | *Clostridium thermocellum* NCIB 10682EL | 1CEN [[18](#_ENREF_18)] | 1CEO [[18](#_ENREF_18)] | Cellobiose | 0.14 |
| Cel5A | *Fervidobacterium nodosum* Rt17-B1 | 3RJX [[19](#_ENREF_19)] | 3RJY [[19](#_ENREF_19)] | Glucose | 0.32 |
| XG5 | *Paenibacillus pabuli* | 2JEP [[20](#_ENREF_20)] | 2JEQ [[20](#_ENREF_20)] | Xyloglucan | 0.22 |
| Cel5G | *Pseudoalteromonas haloplanktis* A23 | 1TVN [[21](#_ENREF_21)] | 1TVP [[21](#_ENREF_21)] | Cellobiose | 0.09 |
| EGCase II | *Rhodococcus* sp. M-777 | 2OSW [[22](#_ENREF_22)] | 2OSY [[22](#_ENREF_22)] | Cellobiose | 0.19 |
| TfManA | *Thermobifida fusca* KW3 | 1BQC [[3](#_ENREF_3)] | 3MAN [[3](#_ENREF_3)] | Mannotriose | 0.19 |
| Cel5A | *Thermobifida fusca* YX | 2CKS [[23](#_ENREF_23)] | 2CKR [[23](#_ENREF_23)] | Cellotetraose | 0.16 |
| Cel5A | *Thermotoga maritima* MSB8 | 3AMC [[24](#_ENREF_24)] | 3AZS [[24](#_ENREF_24)] | Mannotriose | 0.31 |
| TpMan | *Thermotoga petrophila* RKU-1 | 3PZ9 [[25](#_ENREF_25)] | 3PZI [[25](#_ENREF_25)] | Glucose | 0.10 |
| Cel5A | uncultured bacterium | 4HTY [[26](#_ENREF_26)] | 4HU0 [[26](#_ENREF_26)] | Cellotetraose | 0.18 |
| Exg | *Candida albicans* | 1CZ1 [[27](#_ENREF_27)] | 3N9K [[28](#_ENREF_28)] | Laminaritriose | 0.20 |
| CelA;EglA | *Piromyces rhizinflatus* 2301 | 3AYR [[2](#_ENREF_2)] | 3AYS [[2](#_ENREF_2)] | Cellotriose | 0.24 |
| Man5A | *Trichoderma reesei* RUTC-30 | 1QNO [[9](#_ENREF_9)] | 1QNR [[9](#_ENREF_9)] | Mannobiose | 0.23 |

**Materials and Methods**

Structural Alignments

The PDB ids for the selected GH5 structures were obtained from the CAZy database (<http://www.cazy.org/> [[29](#_ENREF_29)]). The unbound and bound structures were aligned using PyMol [[30](#_ENREF_30)].

**References**

1. Blommel PG, Martin PA, Seder KD, Wrobel RL, Fox BG (2009) Flexi vector cloning. Methods Mol Biol 498: 55-73.

2. Tseng CW, Ko TP, Guo RT, Huang JW, Wang HC, et al. (2011) Substrate binding of a GH5 endoglucanase from the ruminal fungus *Piromyces rhizinflata*. Acta Crystallogr Sect F Struct Biol Cryst Commun 67: 1189-1194.

3. Hilge M, Gloor SM, Rypniewski W, Sauer O, Heightman TD, et al. (1998) High-resolution native and complex structures of thermostable beta-mannanase from *Thermomonospora fusca* - substrate specificity in glycosyl hydrolase family 5. Structure 6: 1433-1444.

4. Akita M, Takeda N, Hirasawa K, Sakai H, Kawamoto M, et al. (2004) Crystallization and preliminary X-ray study of alkaline mannanase from an alkaliphilic *Bacillus* isolate. Acta Crystallogr D Biol Crystallogr 60: 1490-1492.

5. Zhao Y, Zhang Y, Cao Y, Qi J, Mao L, et al. (2011) Structural analysis of alkaline beta-mannanase from alkaliphilic *Bacillus* sp. N16-5: implications for adaptation to alkaline conditions. PLoS One 6: e14608.

6. Kumagai Y, Usuki H, Yamamoto Y, Yamasato A, Arima J, et al. (2011) Characterization of calcium ion sensitive region for beta-mannanase from *Streptomyces thermolilacinus*. Biochim Biophys Acta 1814: 1127-1133.

7. Bianchetti CM, Brumm P, Smith RW, Dyer K, Hura GL, et al. (2013) Structure, dynamics and specificity of endoglucanase D from *Clostridium cellulovorans*. J Mol Biol 425: 4267-4285.

8. Hogg D, Woo EJ, Bolam DN, McKie VA, Gilbert HJ, et al. (2001) Crystal structure of mannanase 26A from *Pseudomonas cellulosa* and analysis of residues involved in substrate binding. J Biol Chem 276: 31186-31192.

9. Sabini E, Schubert H, Murshudov G, Wilson KS, Siika-Aho M, et al. (2000) The three-dimensional structure of a *Trichoderma reesei* beta-mannanase from glycoside hydrolase family 5. Acta Crystallogr D Biol Crystallogr 56: 3-13.

10. Kim HW, Ishikawa K (2010) Structure of hyperthermophilic endocellulase from *Pyrococcus horikoshii*. Proteins 78: 496-500.

11. Kim HW, Ishikawa K (2011) Functional analysis of hyperthermophilic endocellulase from *Pyrococcus horikoshii* by crystallographic snapshots. Biochem J 437: 223-230.

12. Baker JO, McCarley JR, Lovett R, Yu CH, Adney WS, et al. (2005) Catalytically enhanced endocellulase Cel5A from *Acidothermus cellulolyticus*. Appl Biochem Biotechnol 121-124: 129-148.

13. Sakon J, Adney WS, Himmel ME, Thomas SR, Karplus PA (1996) Crystal structure of thermostable family 5 endocellulase E1 from *Acidothermus cellulolyticus* in complex with cellotetraose. Biochemistry 35: 10648-10660.

14. Tailford LE, Ducros VM, Flint JE, Roberts SM, Morland C, et al. (2009) Understanding how diverse beta-mannanases recognize heterogeneous substrates. Biochemistry 48: 7009-7018.

15. Shirai T, Ishida H, Noda J, Yamane T, Ozaki K, et al. (2001) Crystal structure of alkaline cellulase K: insight into the alkaline adaptation of an industrial enzyme. J Mol Biol 310: 1079-1087.

16. Dias FM, Vincent F, Pell G, Prates JA, Centeno MS, et al. (2004) Insights into the molecular determinants of substrate specificity in glycoside hydrolase family 5 revealed by the crystal structure and kinetics of *Cellvibrio mixtus* mannosidase 5A. J Biol Chem 279: 25517-25526.

17. Vincent F, Gloster TM, Macdonald J, Morland C, Stick RV, et al. (2004) Common inhibition of both beta-glucosidases and beta-mannosidases by isofagomine lactam reflects different conformational itineraries for pyranoside hydrolysis. Chembiochem 5: 1596-1599.

18. Dominguez R, Souchon H, Lascombe M, Alzari PM (1996) The crystal structure of a family 5 endoglucanase mutant in complexed and uncomplexed forms reveals an induced fit activation mechanism. J Mol Biol 257: 1042-1051.

19. Zheng B, Yang W, Zhao X, Wang Y, Lou Z, et al. (2012) Crystal structure of hyperthermophilic endo-beta-1,4-glucanase: implications for catalytic mechanism and thermostability. J Biol Chem 287: 8336-8346.

20. Gloster TM, Ibatullin FM, Macauley K, Eklof JM, Roberts S, et al. (2007) Characterization and three-dimensional structures of two distinct bacterial xyloglucanases from families GH5 and GH12. J Biol Chem 282: 19177-19189.

21. Violot S, Aghajari N, Czjzek M, Feller G, Sonan GK, et al. (2005) Structure of a full length psychrophilic cellulase from *Pseudoalteromonas haloplanktis* revealed by X-ray diffraction and small angle X-ray scattering. J Mol Biol 348: 1211-1224.

22. Caines ME, Vaughan MD, Tarling CA, Hancock SM, Warren RA, et al. (2007) Structural and mechanistic analyses of endo-glycoceramidase II, a membrane-associated family 5 glycosidase in the Apo and GM3 ganglioside-bound forms. J Biol Chem 282: 14300-14308.

23. Berglund GI, Gualfetti PJ, Requadt C, Gross LS, Bergfors T, et al. (2007) PDB ID: 2CKR and 2CKS, The Crystal Structure of the Catalytic Domain of Thermobifida Fusca Endoglucanase Cel5A in Complex with Cellotetraose.

24. Wu TH, Huang CH, Ko TP, Lai HL, Ma Y, et al. (2011) Diverse substrate recognition mechanism revealed by *Thermotoga maritima* Cel5A structures in complex with cellotetraose, cellobiose and mannotriose. Biochim Biophys Acta 1814: 1832-1840.

25. Santos CR, Meza AN, Paiva JH, Silva JC, Ruller R, et al. (2011) PDB ID: 3PZ9 and 3PZI, Structural characterization of a novel hyperthermostable endo-1,4-beta-D-mannanase from Thermotoga petrophila RKU-1.

26. Zhuang N, Lee KH (2012) PDB ID: 4HTY and 4HUO, Crystal structure and substrate-binding mode of cellulase Cel5A from a metagenome library.

27. Cutfield SM, Davies GJ, Murshudov G, Anderson BF, Moody PC, et al. (1999) The structure of the exo-beta-(1,3)-glucanase from *Candida albicans* in native and bound forms: relationship between a pocket and groove in family 5 glycosyl hydrolases. J Mol Biol 294: 771-783.

28. Patrick WM, Nakatani Y, Cutfield SM, Sharpe ML, Ramsay RJ, et al. (2010) Carbohydrate binding sites in *Candida albicans* exo-beta-1,3-glucanase and the role of the Phe-Phe 'clamp' at the active site entrance. FEBS J 277: 4549-4561.

29. Aspeborg H, Coutinho PM, Wang Y, Brumer H, 3rd, Henrissat B (2012) Evolution, substrate specificity and subfamily classification of glycoside hydrolase family 5 (GH5). BMC Evol Biol 12: 186.

30. Delano WL (2002) The PyMOL Molecular Graphics System. Delano Scientific LLC, San Carlos, CA, USA.
